# Single-Nucleotide Resolution Mapping of *N*^6^-Methyladenine in Genomic DNA

**DOI:** 10.1021/acscentsci.3c00481

**Published:** 2023-08-28

**Authors:** Cheng-Jie Ma, Gaojie Li, Wen-Xuan Shao, Yi-Hao Min, Ping Wang, Jiang-Hui Ding, Neng-Bin Xie, Min Wang, Feng Tang, Yu-Qi Feng, Weimin Ci, Yinsheng Wang, Bi-Feng Yuan

**Affiliations:** † School of Public Health, Department of Radiation and Medical Oncology, Zhongnan Hospital of Wuhan University, Wuhan University, Wuhan 430071, China; ‡ Sauvage Center for Molecular Sciences, Department of Chemistry, Wuhan University, Wuhan 430072, China; § Department of Chemistry, University of California, Riverside, Riverside, California 92521-0403, United States; ∥ Key Laboratory of Genomics and Precision Medicine, and China National Center for Bioinformation, Beijing Institute of Genomics, Chinese Academy of Sciences, Beijing 100101, China; ⊥ University of Chinese Academy of Sciences, Beijing 100049, China

## Abstract

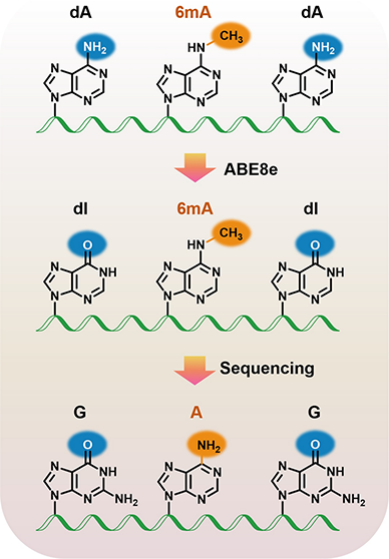

*N*^6^-Methyladenine (6mA) is a naturally occurring DNA modification in both prokaryotes and eukaryotes. Herein, we developed a deaminase-mediated sequencing (DM-seq) method for genome-wide mapping of 6mA at single-nucleotide resolution. The method capitalizes on the selective deamination of adenine, but not 6mA, in DNA mediated by an evolved adenine deaminase, ABE8e. By employing this method, we achieved genome-wide mapping of 6mA in *Escherichia coli* and in mammalian mitochondrial DNA (mtDNA) at single-nucleotide resolution. We found that the 6mA sites are mainly located in the GATC motif in the *E. coli* genome. We also identified 17 6mA sites in mtDNA of HepG2 cells, where all of the 6mA sites are distributed in the heavy strand of mtDNA. We envision that DM-seq will be a valuable tool for uncovering new functions of 6mA in DNA and for exploring its potential roles in mitochondria-related human diseases.

## Abstract 2

We report a method for mapping *N*^6^-methyladenine (6ma) in DNA at single-nucleotide resolution, which may be useful for revealing new functions of 6mA in DNA and its roles in diseases.

## Introduction

Aside from the four canonical nucleobases, a number of modified nucleobases have been identified in genomes in a variety of organisms.^1^ Among them, 5-methylcytosine (5mC) is the best-characterized epigenetic modification that plays critical roles in a wide range of biological processes in mammals.^2^

6mA is a natural DNA modification that functions primarily in restriction-modification (R-M) systems in prokaryotes.^3^ 6mA is also involved in DNA mismatch repair and gene regulatory processes in *Escherichia coli* and other bacterial species.^4^ In addition, recent studies uncovered the existence and revealed the genome-wide distribution of 6mA in the DNA of different eukaryotes, including mammals and plants.^5−14^ Although the levels of 6mA are high in the genomes of some invertebrates, its levels are generally low in those of vertebrates and mammals, ranging from a few to tens of parts per million (ppm) of the total adenines.^7−10,15^ 6mA was suggested to assume important roles in embryonic development,^6,7^ tumorigenesis,^9^ neuropsychiatric disorders,^16^ and mitochondrial stress adaptation.^17^ However, the results from metabolic isotopic labeling coupled with mass spectrometric analysis revealed that, in some cases, 6mA in genomic DNA originates from *N*^6^-methyladenosine in RNA, which is processed through a nucleotide-salvage pathway and then misincorporated into DNA by DNA polymerases.^18,19^ These studies argue against 6mA being a DNA epigenetic mark in mammalian genomes. Nevertheless, Hao et al.^20^ recently reported that 6mA is enriched in mammalian mitochondrial DNA (mtDNA) and regulates mitochondrial transcription, replication, and activity. The level of 6mA in mtDNA can be 1300-fold higher than that in total DNA.^20^

Revealing the functions of 6mA requires precise mapping of its locations in DNA. Direct sequencing of 6mA has been challenging because this modified nucleobase is “silent” in conventional high-throughput sequencing. In recent years, several strategies have been developed to map 6mA in DNA. Immunoprecipitation followed by sequencing, 6mA DIP-seq,^21−23^ has been developed to map 6mA in genomes of mammalian cells; the method, however, suffers from low resolution. *Dpn* I-assisted 6mA sequencing (DA-6mA-seq) employs *Dpn* I to cleave methylated adenine sites (5′-G6mATC-3′) in duplex DNA.^24^ The cleavage pattern is employed to map 6mA sites in DNA but is limited to a small subset of adenines located at GATC sites. 6mA cross-linking exonuclease sequencing (6mACE-seq) utilizes cross-linking with 6mA-specific antibodies to protect 6mA-DNA fragments from subsequent exonuclease digestion.^25^ The 6mA-CLIP-exo method combines immunoprecipitation, photo-cross-linking, and exonuclease digestion to map 6mA sites, where the method provided a mapping resolution of ∼30 nucleotides.^11^ Single-molecule real-time (SMRT) sequencing has also been employed to detect 6mA in mouse embryonic stem cells (mESCs) and plant genomes.^10,26^ Nonetheless, recent reports showed that SMRT sequencing exhibits high false-positive rates and overestimates the amount of 6mA measured in eukaryotic genomes.^27,28^ In addition, Kong et al.^29^ recently reported very low levels of 6mA in peripheral blood mononuclear cells (PBMCs) and undetectable levels of 6mA in many other cells or tissues by the SMRT sequencing-based 6mASCOPE method. Together, controversial results have been obtained for the presence of 6mA in mammalian genomes, and robust sequencing methods are needed to resolve these controversies and facilitate functional studies of the 6mA methylome.

Herein, we report a deaminase-mediated sequencing (DM-seq) method for mapping 6mA in DNA at single-nucleotide resolution. ABE8e was initially evolved from an *E. coli* tRNA adenine deaminase, which, upon fusion with the Cas9 protein, could catalyze targeted deamination of dA to 2′-deoxyinosine (dI) in DNA and is utilized as the DNA adenine base editor to convert A:T to G:C base pairs.^30^ We reason that the ABE8e protein should catalyze the deamination of adenines in DNA and that the presence of a methyl group at the *N*^6^ position of adenine may prohibit the ABE8e-mediated deamination of 6mA, which could be harnessed to map 6mA in DNA at single-nucleotide resolution.

## Results and Discussion

**Figure 1:**
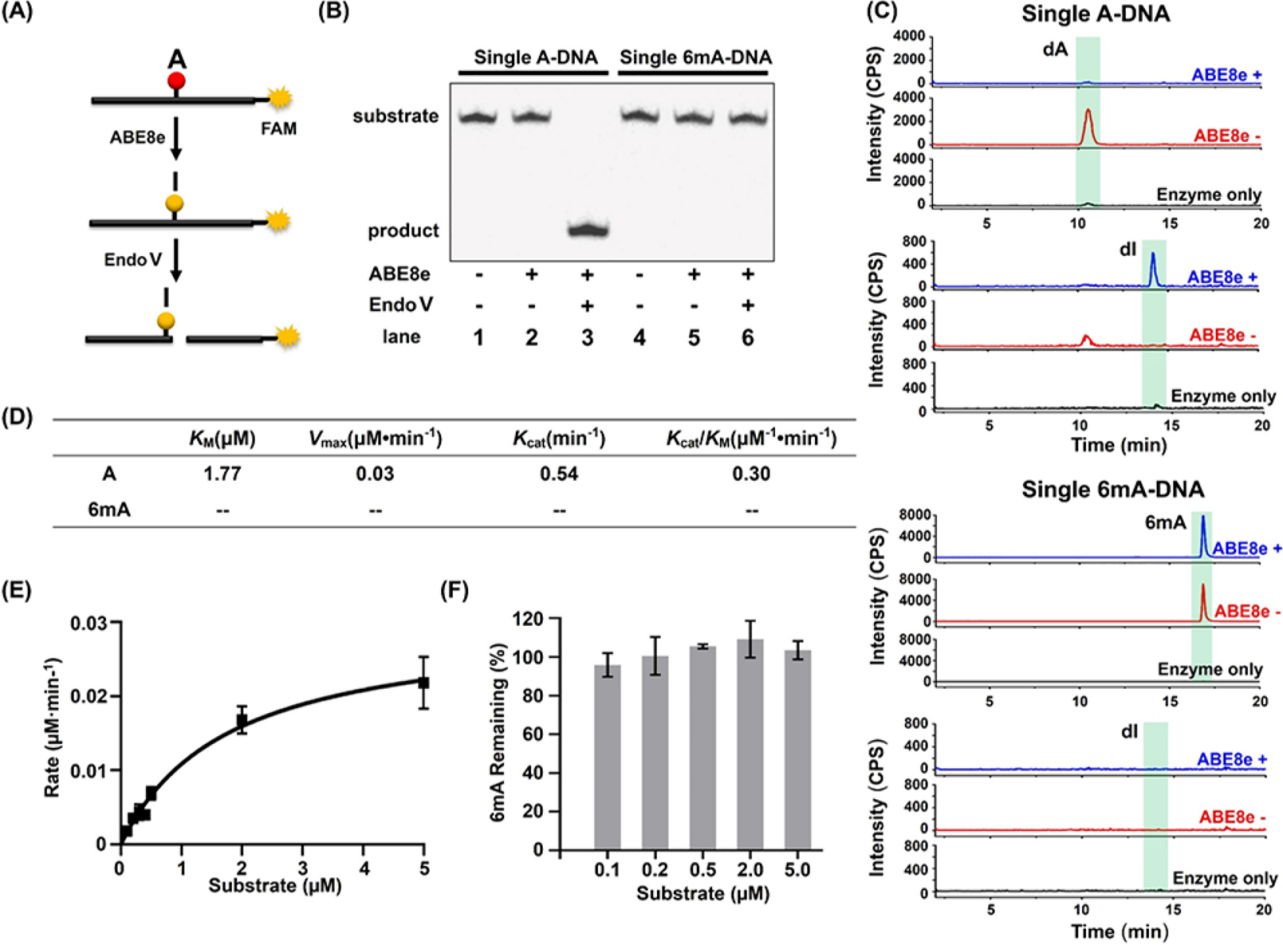
Evaluation of the ABE8e-mediated deamination of dA and 6mA in DNA by ABE8e. (A) Workflow of the Endo V cleavage assay to assess the deamination of dA and 6mA. The dI formed from deamination of dA can be cleaved by Endo V. (B) Analysis of the 60-mer single A-DNA and single 6mA-DNA after ABE8e treatment at 37 °C for 30 min. The resulting DNA was incubated with Endo V at 37 °C for 1 h followed by polyacrylamide gel electrophoresis analysis. (C) LC-ESI-MS/MS analysis of dA, dI, and 6mA nucleosides from the 60-mer single A-DNA and single 6mA DNA with or without ABE8e treatment. (D) Steady-state kinetic parameters for deamination of dA or 6mA in DNA by ABE8e. (E) Michaelis–Menten plot showing the rate of ABE8e-mediated deamination versus the concentrations of the 60-mer single A-DNA. Data were fit with the Michaelis–Menten equation. (F) LC-ESI-MS/MS analysis of the remaining percentage of 6mA from different amounts of the 60-mer single 6mA DNA after ABE8e treatment, at 2.8 μM for 30 min.

We first expressed and purified recombinant ABE8e protein (Figure S1) and used a 5′-FAM-labeled 60-mer single-stranded DNA that contains a single dA or 6mA site (Table S1) to assess the ABE8e-catalyzed deamination of dA and 6mA. Since the deamination of dA by ABE8e yields a dI that can be cleaved by Endo V (Figure 1A), we developed the Endo V cleavage assay to examine the deamination of dA and 6mA in DNA (Figure S2), and the results showed that A-DNA could be converted to I-DNA upon ABE8e treatment and then cleaved by Endo V to generate a strand break at the initial A site (Figure 1B, lane 3). However, after ABE8e treatment, 6mA-DNA fully resisted Endo V cleavage, indicating that 6mA could not be deaminated by ABE8e (Figure 1B, lane 6). The A-DNA and 6mA-DNA were mixed at different molar ratios, followed by treatment with ABE8e and Endo V. The results showed that the cleaved fractions were proportional to the percentages of A-DNA in the mixture (Figure S3), suggesting that the level of dA in DNA can be quantitatively determined with the deamination assay. We also conducted the deamination reaction for different time intervals (0, 1, 5, 10, and 30 min), and our results showed that complete conversion of dA to dI could be achieved within 10 min (Figure S4). The specific activity of ABE8e was estimated to be 7 × 10^–4^ μmol min^–1^ mg^–1^.

We further employed LC-ESI-MS/MS to evaluate the ABE8e-catalyzed deamination of dA and 6mA in DNA (mass spectrometry parameters are given in Table S2). The results showed that dA in a single A-DNA was undetectable following ABE8e treatment; in the meantime, the deaminated product (i.e., dI) was readily detected (Figure 1C). In contrast, no dI signal was observed in the single 6mA-DNA sample after ABE8e treatment, and the signal intensity of 6mA was comparable between the samples with and without ABE8e treatment (Figure 1C). As expected, ABE8e treatment did not alter the levels of other nucleosides (i.e., dG, dC, and dT) in single A-DNA or 6mA-DNA (Figure S5).

We next measured the deamination efficiency of ABE8e toward dA and 6mA by using a steady-state kinetic study. The results revealed a *k*_cat_/*K*_M_ value of 0.3 μM^–1^ min^–1^ with the single A-DNA substrate (Figure 1D,E). However, due to the extremely low activity of ABE8e toward 6mA, the kinetic parameters could not be obtained using a single 6mA-DNA as the substrate. The LC-ESI-MS/MS analysis showed that ABE8e treatment did not lead to any appreciable change in the percentage of 6mA using different amounts of the single 6mA-DNA substrate (Figure 1F). In addition, 6mA is resistant to deamination even with a 10× higher concentration of ABE8e (28 μM) or an extended incubation time (4 h, Figure S6). Collectively, the results from the Endo V cleavage assay and LC-ESI-MS/MS analysis revealed that ABE8e could specifically and efficiently deaminate dA, but not 6mA or other nucleobases in DNA.

**Figure 2:**
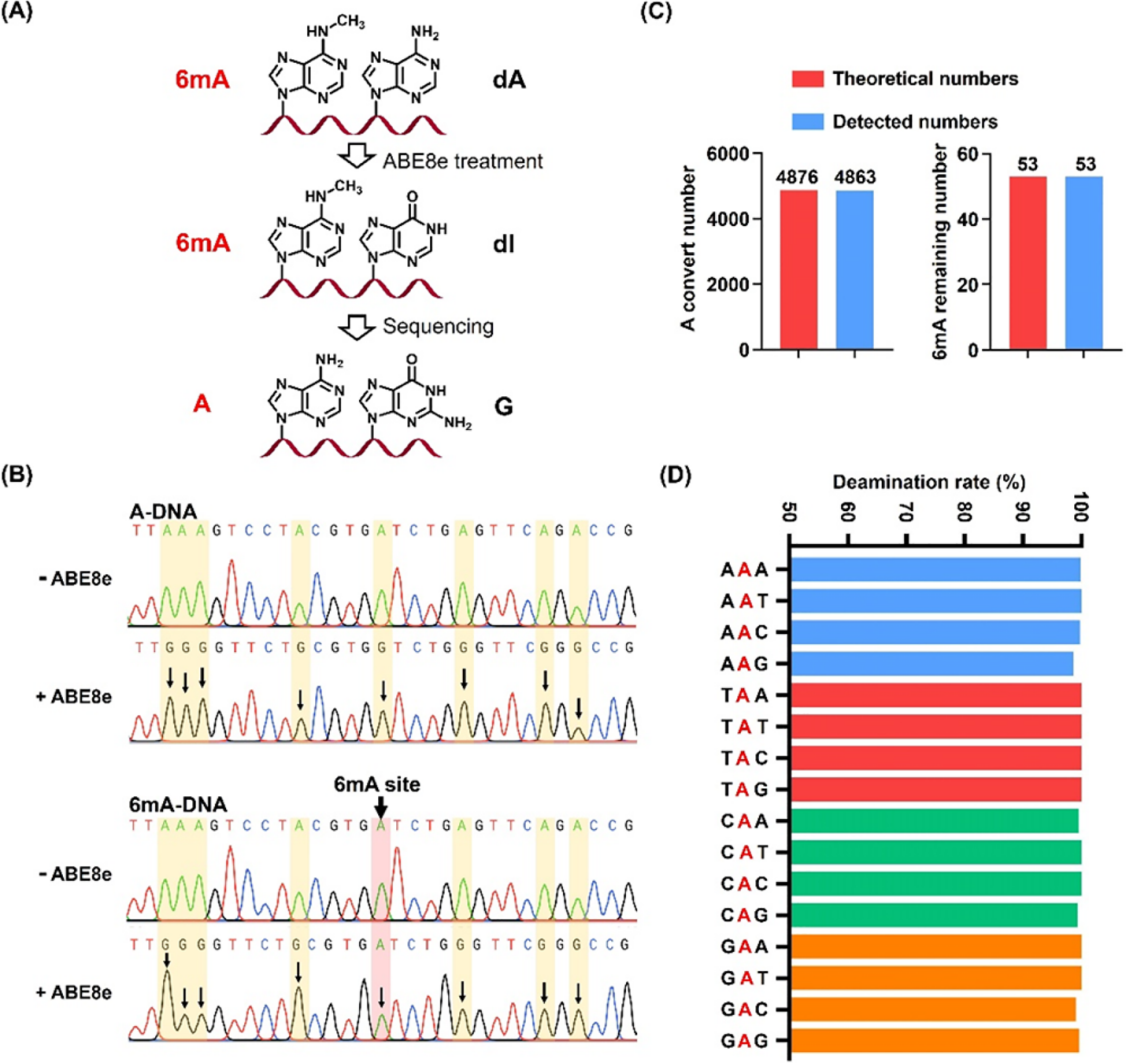
Evaluation of the DM-seq method by sequencing. (A) Schematic illustration of deaminase-mediated mapping of 6mA in DNA. (B) Sanger sequencing of 314-bp A-DNA and 314-bp 6mA-DNA with or without ABE8e treatment. (C) Quantitative measurements of A-to-G conversion rates and evaluation of the 6mA readout in the 314-bp 6mA-DNA cloned after ABE8e treatment followed by colony sequencing. Fifty-three colonies were picked for sequencing. (Figure S8). (D) Statistical analysis of the deamination rate of dA with different flanking nucleobases of 314-bp 6mA-DNA by colony sequencing.

We next established a deaminase-mediated sequencing (DM-seq) method by using 314-bp A-DNA and 314-bp 6mA-DNA, the latter of which carries a single 6mA (Figure 2A, Table S1, and Figure S7). The 314-bp A-DNA and 6mA-DNA were treated with ABE8e followed by PCR amplification and Sanger sequencing. All of the dAs in the 314-bp A-DNA and 314-bp 6mA-DNA were read as G after ABE8e treatment (Figure 2B), whereas the 6mA in 314-bp 6mA-DNA was still read as A following ABE8e treatment (Figure 2B). The sequencing results indicate that the DM-seq method is capable of mapping 6mA in DNA at single-nucleotide resolution by virtue of the specific and efficient ABE8e-mediated deamination of dA, but not 6mA in DNA.

We also cloned the PCR products, transformed them into *E. coli*, and conducted colony sequencing to evaluate quantitatively the A-to-G conversion rate. A total of 53 colonies were randomly picked for sequencing. Statistical analysis of the sequencing results showed that the 6mA sites in all 53 sequenced colonies were read as A (Figure S8). However, almost all of the dAs in the 314-bp 6mA-DNA were completely deaminated and read as G (Figure S8). A total of 4863 dA sites from 53 clones were read as G after ABE8e treatment, with the overall A-to-G conversion rate being 99.7% (Figure 2C and Figure S8). We also analyzed the deamination efficiencies of dA in different sequence contexts. The results demonstrated that dAs in all of the sequence contexts were efficiently deaminated, with the deamination efficiency being greater than 98.7% (Figure 2D and Table S3). Together, these results underscored that the DM-Seq method exhibits a very low false-positive rate and displays no sequence bias in detecting 6mA sites.

We also spiked a 60-mer A-DNA with different percentages (i.e., 0%, 5%, 10%, 20%, 50%, and 100%) of 60-mer 6mA-DNA in the same sequence (Table S1) and evaluated the readouts of A and G from DM-seq. The results showed that the percentages of A reads from the initial 6mA site are proportional to the percentages of spiked-in 6mA-DNA, and excellent linearity was obtained with the slope and *R*^2^ being 1.002 and 0.9948, respectively (Figure S9). The results showed that DM-seq provides robust quantitative assessment of a low stoichiometric amount of 6mA. Moreover, we synthesized a 60-mer DNA harboring an adenine flanked with two 6mA sites in the sequence context of AA6mAA6mAAA (Table S1 and Figure S10A) and subjected the DNA to DM-seq analysis. The colony sequencing result demonstrated that all A sites in this region were efficiently deaminated and read as G in sequencing, with the deamination rate of A being 99.6% (Figure S10B,C). In contrast, both 6mA sites in this region resisted deamination and still read as A, with the deamination rate being 0% (Figure S10B,C). Collectively, the above results revealed the distinct coding properties of dA and 6mA in DNA after ABE8e treatment, suggesting that DM-seq is amenable for mapping 6mA in DNA at single-base resolution.

Recently, nitrite was utilized for mapping 6mA, where nitrite could induce the deamination of adenine, but not 6mA in DNA.^31,32^ For comparison, we also carried out nitrite treatment and sequencing analysis. To this end, we treated the aforementioned 314-bp DNA with nitrite by following previously described conditions and analyzed the resulting samples by Sanger sequencing.^32^ The sequencing results were messy (Figure S11A).

We next performed colony sequencing for the nitrite-treated samples. The results showed that some A and C were deaminated and read as G and T, respectively (Figure S11B); however, incomplete deamination was observed for both A and C. The results are consistent with previous reports that nitrite treatment could induce the deamination of both A and C.^31,32^ In addition, nitrite treatment may also result in the deamination of G to produce xanthine, which may lead to G to A conversion.^33^ Therefore, nitrite treatment leads to complicated sequencing results, where the data analysis entails a sophisticated bioinformatic workflow.

**Figure 3:**
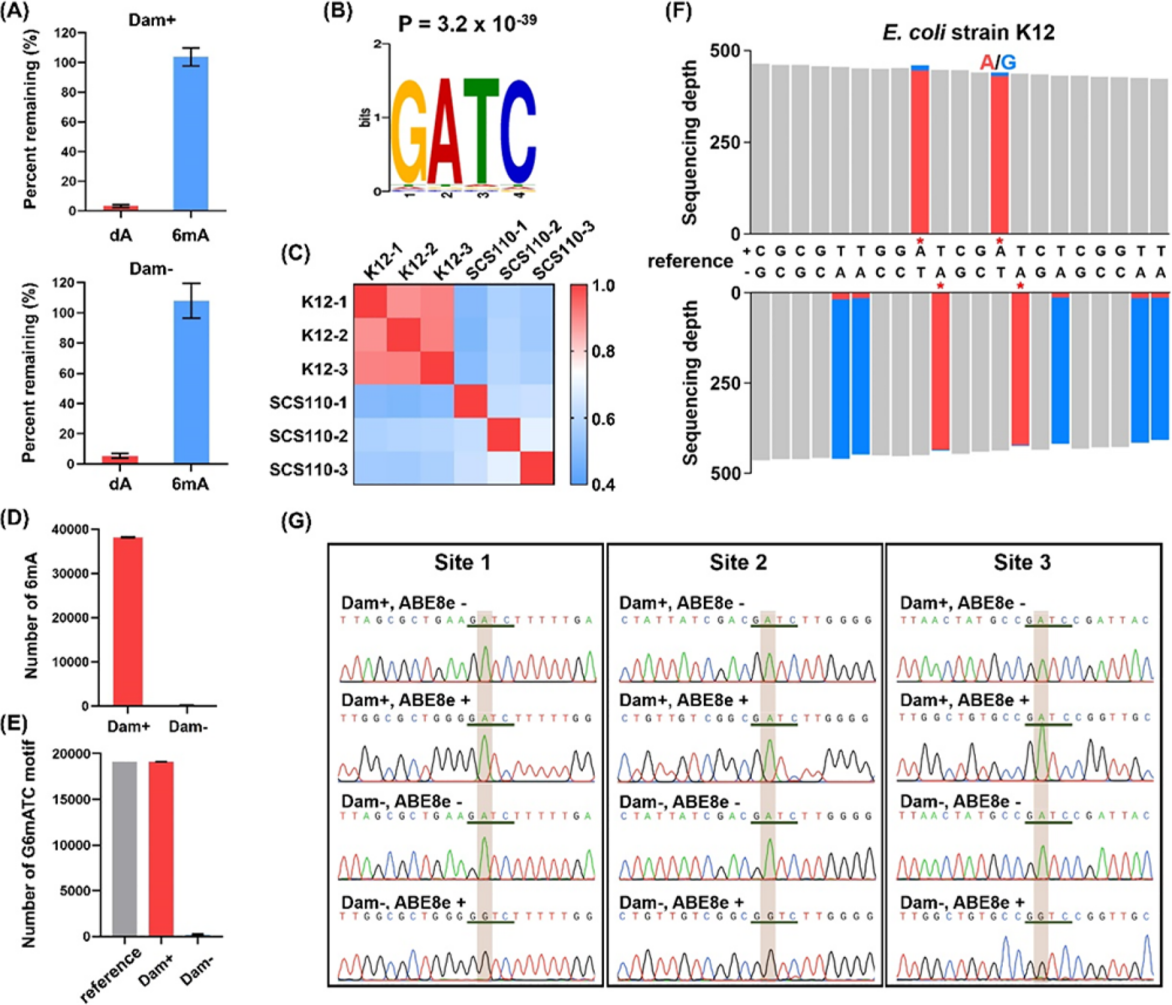
Genome-wide mapping of 6mA in *E. coli* by DM-seq. (A) LC-ESI-MS/MS analysis of the remaining percentages of dA and 6mA in genomic DNA of wild-type and *dam*-deficient *E. coli* strains after ABE8e treatment. Each percentage is relative to the amount in the respective strains prior to ABE8e treatment. (B) Motif sequence profile and sequence conservation analysis. (C) Heat map showing the correlation of the identified 6mA sites in different replicates. (D) Numbers of 6mA sites identified in the genomes (from both DNA strands) of wild-type and *dam*-deficient *E. coli* strains. (E) Theoretical numbers of GATC motifs (gray column) and numbers of identified G6mATC motifs in the genomes of wild-type (red column) and *dam*-deficient (blue column) *E. coli* strains. G6mATC from duplex DNA was recognized as one site. (F) Representative view of 6mA sites in the genome of the wild-type *E. coli* strain (position: 98809 to 98829). Red asterisks denote 6mA sites. Red and blue columns represent the number of reads with A and G at the indicated sites, respectively. (G) Sanger sequencing of three different 6mA sites with or without ABE8e treatment in the genomes of wild-type and *dam*-deficient *E. coli* strains.

6mA is a naturally occurring DNA modification that is conserved in prokaryotes and participates in the R-M systems.^3^ Next we used the established DM-seq method to map 6mA in wild-type *E. coli* strain K12 and *dam*-deficient *E. coli* strain SCS110. LC-ESI-MS/MS analysis revealed no apparent change in 6mA in genomic DNA from the K12 and SCS110 strains upon ABE8e treatment (Figure 3A and Figure S12), substantiating that 6mA in *E. coli* DNA is resistant to ABE8e-mediated deamination.

We then applied the DM-seq method to map 6mA in the *E. coli* genome by using high-throughput sequencing (Figure S13 and Table S4). A 328-bp 6mA-DNA (Table S1) was spiked-in as the control in the library construction using 2.8 μM ABE8e for 4 h. The unbiased analysis of the spiked-in 328-bp 6mA-DNA showed that the average deamination rate (A-to-G conversion) of dA was 93.4%, whereas over 97.9% of 6mA remained intact after ABE8e treatment (Table S5). Three biological replicates were measured, and 18.6, 15.9, and 19.9 million clean reads were obtained for the three replicates of DNA isolated from the K12 strain. Similarly, 18.3, 17.1, and 15.6 million clean reads were obtained for the three replicates of DNA from the SCS110 strain (Table S6). A total of 71% of the clean reads from each replicate could be mapped to the *E. coli* reference genome, yielding an average sequencing depth of 187× for the genome (Table S6).

Motif analysis showed that 6mA occurred predominantly in the GATC sequence motif (*p* = 3.2 × 10^–39^) (Figure 3B). The identified 6mA sites in the K12 strain were highly correlated among the three replicates (Figure 3C). In contrast, the 6mA sites identified in the *dam*-deficient SCS110 strain were more randomly distributed (Figure 3C). Approximately 38 000 6mA sites were detected in the wild-type K12 strain. This is expected as the DNA was extracted from a stationary-phase culture, in which GATC sites should be fully methylated,^34^ and the K12 genome contains 38 248 GATC sites (accession no. NC_000913.3). By contrast, a very small number of 6mA sites (∼200) were observed in the *dam*-deficient SCS110 strain (Figure 3D). Quantitative analysis showed that adenine in the GATC motif was predominantly methylated (99.9%) in the wild-type *E. coli* genome (Figure 3E). In contrast, a majority of adenines in the GATC motif in *dam*-deficient *E. coli* strain SCS110 were unmethylated (Figure 3E). Figure 3F and Figure S14 show representative maps of 6mA sites in the region of 98 809 to 98 829 in the genomes of wild-type (K12) and *dam*-deficient (SCS110) strains, respectively. All of the identified G6mATC motifs from three replicates of the K12 strain are summarized in the circos plot (Figure S15).

We further confirmed three different 6mA sites in the GATC motif from *E. coli* cells by Sanger sequencing. All of the dAs in the GATC motif were read as A in DNA isolated from the K12 strain; however, all those dAs not in the GATC motif were read as G after ABE8e treatment (Figure 3G). On the other hand, all of the dA in DNA isolated from the *dam*-deficient SCS110 strain, regardless of whether it resides in the GATC motif, was read as G after ABE8e treatment (Figure 3G). The Sanger sequencing results further confirmed the accuracy of the DM-seq method in mapping 6mA.

**Figure 4:**
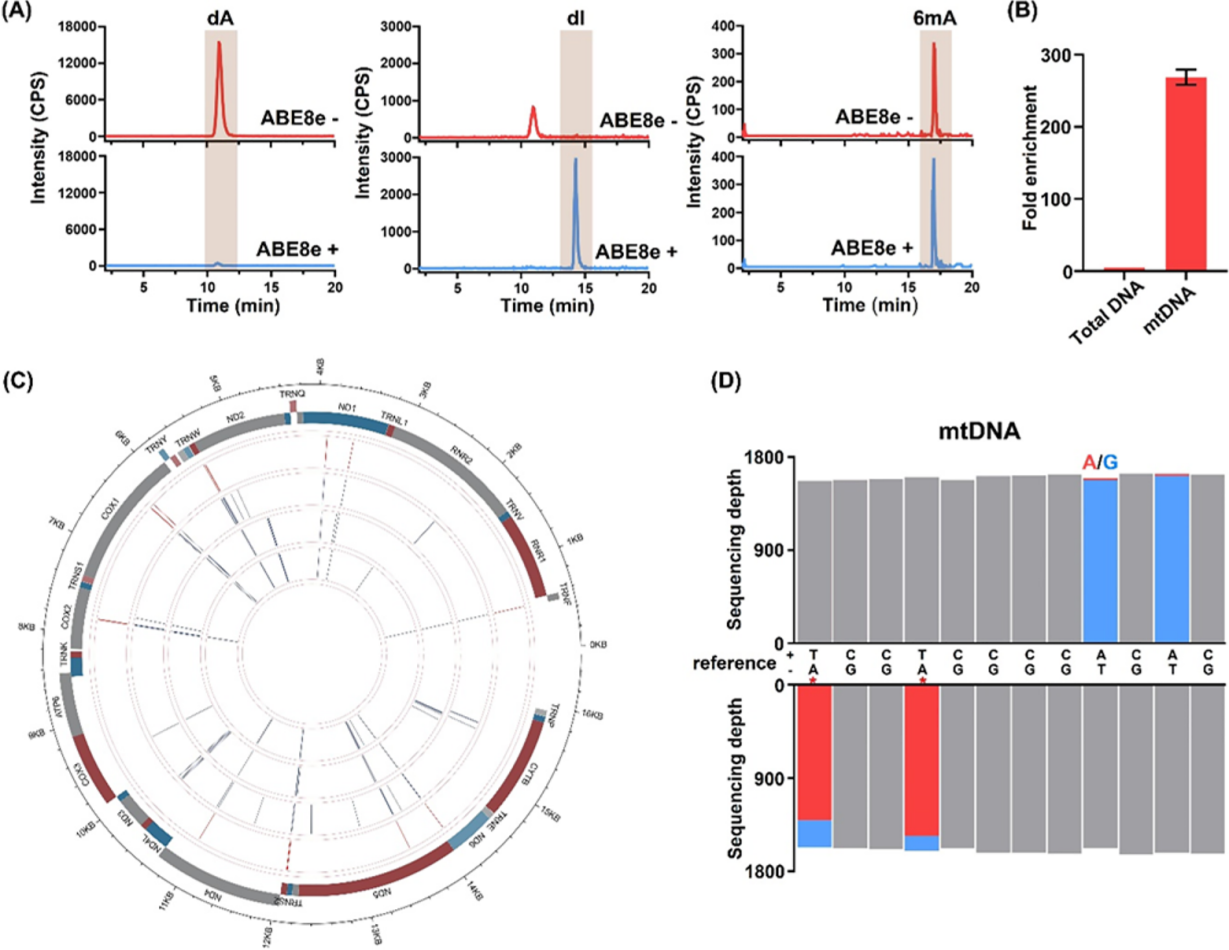
Genome-wide mapping of 6mA in mtDNA by DM-seq. (A) LC-ESI-MS/MS for evaluating the levels of dA and 6mA in human HepG2 mtDNA after ABE8e treatment. ABE8e treatment led to the production of dI from dA. (B) Evaluation of the enrichment fold for mtDNA by qPCR. (C) Circos plot showing the distribution of 6mA sites across human HepG2 mtDNA. The inner three circles represent three biological replicates; the outer circle represents 6mA sites (red bars). (D) Representative view of 6mA sites in human HepG2 mtDNA (positions 5443 to 5454). Red asterisks denote 6mA sites. Red and blue columns represent the respective numbers of A and G reads at the indicated positions.

A previous study showed that 6mA was more abundant in mtDNA than in the genomic DNA of mammals.^20^ Thus, we also examined the 6mA sites in the mtDNA of human HepG2 cells. As expected, LC-ESI-MS/MS analysis showed that ABE8e treatment led to the efficient conversion of dA to dI; however, the level of 6mA was similar with or without ABE8e treatment (Figure 4A). Real-time qPCR analysis showed a 270-fold enrichment of mtDNA over total DNA with the current mtDNA isolation method (Figure 4B and Figure S16). In addition, mtDNA accounted for ∼50% of total mapped reads from sequencing analysis (Figure S17).

We next profiled 6mA in mtDNA using DM-seq. The nuclear DNA segments were filtered out to ensure that the reads are exclusively mapped to mtDNA. Three biological replicates were sequenced, and only those 6mA sites detected in all three replicates with the methylation rate being ≥0.7 are considered high-fidelity 6mA sites. Seventeen such 6mA sites were identified, and strikingly all of these sites are situated in the heavy strand of mtDNA (Table S7). It is worth noting that none of these 6mA sites is located in the GATC sequence context. Furthermore, approximately 65% of the 6mA sites were located in the same regions of human HepG2 mtDNA reported by a previous study using antibody enrichment-based sequencing.^20^ Moreover, we employed Sanger sequencing to validate some 6mA sites identified by the DM-seq method. The results demonstrated that 6mA sites detected by Sanger sequencing are consistent with those identified by DM-seq (Figure S18). We summarized all of the identified 6mA sites from three replicates in the circos plot (Figure 4C). Shown in Figure 4D is a representative map of 6mA sites in the region between 5443 and 5454 of human mtDNA. Collectively, these results indicated that the DM-seq method is capable of mapping 6mA in mtDNA.

Epigenetic modifications in DNA have been demonstrated to play critical roles in regulating gene expression. 5mC is the most extensively studied epigenetic marker in mammals. Apart from 5mC, 6mA is prevalent in bacteria, playing crucial roles in R-M systems as well as in DNA replication and repair. Recent studies uncovered the presence of 6mA in eukaryotic DNA and mammalian mtDNA,^5−14,20^ which sheds light on the potential functions of 6mA in diverse eukaryotes.

Unveiling the functions of 6mA necessitates precise mapping of 6mA in the genomes of living organisms. Here we developed the DM-seq method for mapping 6mA in DNA at single-nucleotide resolution. This method capitalizes on the selective deamination of adenine, but not 6mA in DNA by using an evolved adenine deaminase, ABE8e. This observation is consistent with an earlier study showing the resistance of 6mA toward deamination.^35^ We found that under optimized conditions, ABE8e allowed for a 99.7% deamination efficiency of unmethylated adenine in DNA (evaluated by colony sequencing, Figure 2C), and minimal deamination was observed for 6mA (Figure 2C), which forms the basis for the DM-seq method. Although the deamination of dA is not 100%, the undeaminated adenines are randomly distributed in DNA, which would not affect 6mA identification by high-throughput sequencing with a high sequencing depth (average sequencing depth of 187× for *E. coli* genomes and 1889× for human mtDNA, Table S6).

Compared with other antibody- and restriction cleavage-based 6mA mapping methods, DM-seq exhibits several significant advantages. First, the DM-seq method is capable of single-nucleotide resolution mapping of 6mA in DNA. Some previously developed methods for mapping 6mA utilized antibody-based enrichment and sequencing, which do not yield information about the location of 6mA at single-nucleotide resolution. Second, the principle of DM-seq is straightforward, and the analytical procedure is simple. In DM-seq, the deamination reaction is carried out under mild conditions where DNA is not susceptible to degradation. Additionally, there is no need for UV- or chemical-reaction-based cross-linking during library construction. Thus, only nanogram quantities of DNA are required for 6mA mapping analysis, and the entire workflow for sequencing library construction could be completed within a single day. Third, DM-seq exhibits a low false-positive rate and no sequence bias in 6mA mapping, where we did not observe any bias in sequence context for the ABE8e-mediated deamination of adenine in DNA. Thus, unlike the restriction enzyme-based cleavage strategy that can only map 6mA in a given sequence context in DNA, the DM-seq method offers precise and comprehensive mapping of 6mA in any sequence contexts in the mammalian genome.

It is also worth comparing DM-seq with the previously published 6mA mapping method relying on nitrite-based deamination. In this vein, nitrite treatment shares the common attribute of being able to deaminate adenine in DNA to yield inosine, whereas 6mA is converted to *N*^6^-nitroso-6mA (6mA-NO) upon nitrate exposure. Hence, nitrite treatment also offers single-nucleotide-resolution mapping of 6mA in DNA. However, nitrite treatment differs from ABE8e-mediated deamination in several important aspects. First, cytosine and, to a lesser degree, guanine in DNA are also susceptible to nitrite-mediated deamination. Hence, the method entails a more sophisticated bioinformatics workflow for calling the 6mA site. Nevertheless, nitrite treatment also provides an opportunity for mapping, apart from 6mA, *N*^4^-methylcytosine and 5-methylcytosine in DNA, as reported previously.^32^ Second, compared with nitrite-mediated deamination, ABE8e provides a more complete conversion of adenine in DNA to inosine. This difference renders the ABE8e-based method more amenable for mapping those 6mA sites with a low methylation stoichiometry. Third, ABE8e-catalyzed enzymatic deamination can be conducted under mild conditions, where no appreciable degradation of DNA was observed; nitrite treatment, however, gives rise to a substantial degradation of DNA. Hence, DM-seq has a unique advantage of being able to map 6mA with a low quantity of input DNA. Fourth, DM-seq requires the preparation of recombinant ABE8e protein, whereas nitrite is commercially available and is relatively inexpensive. Thus, the cost for sample pretreatment is lower for the nitrite-based method.

By using DM-seq, we mapped the 6mA sites in the *E. coli* genome and mtDNA isolated from HepG2 cells. As expected, almost all of the adenines in the GATC motif are heavily methylated in the K12 wild-type *E. coli* strain; in contrast, the number of 6mA sites and the methylation level at each 6mA site are greatly diminished in the *dam*-deficient SCS110 strain. Moreover, we identified 17 6mA sites in human mtDNA isolated from HepG2 cells, and strikingly all of the 6mA sites are located in the heavy strand. It is not clear why all of the 6mA sites are situated on one strand of the mitochondrial genome or how that pattern is maintained during mtDNA replication.

The DM-seq method provides a useful tool for profiling 6mA in mtDNA, which may promote the study of mitochondrial evolution. Additionally, the DM-seq method can also be harnessed to profile the tissue-specific 6mA landscape of human mtDNA, which should expedite deciphering 6mA functions in mitochondria-related human diseases. In this respect, the 6mA level was shown to be substantially elevated in cells under hypoxia.^20^ Pathological conditions and/or environmental exposure may give rise to a loss of redox homeostasis,^36^ which may result in an altered 6mA level in mtDNA. The DM-seq method should be amenable for elucidating pathological mechanisms and mechanisms of toxicity associated with environmental exposure. The method also holds potential for the medical diagnosis of human disease. Furthermore, by combining with antibody-based 6mA enrichment, DM-seq may constitute a promising method for mapping 6mA in organisms containing only a part per million level of 6mA in their genomes.

Together, DM-seq is a straightforward and facile method for the precise mapping of 6mA sites at single-nucleotide resolution. It can be envisaged that the DM-seq method will be valuable in uncovering new functions of 6mA in living organisms in the future.

## Experimental Section

### Chemicals and Reagents

The 60-mer single A-containing DNA (single A-DNA), single 6mA-containing DNA (single 6mA-DNA), and single I-containing DNA (single I-DNA) were purchased from Sangon Biotech (Shanghai, China). The sequences of these oligonucleotides are listed in Table S1. 2′-Deoxycytidine (dC), 2′-deoxyguanosine (dG), 2′-deoxyadenosine (dA), thymidine (dT), 2′-deoxyinosine (dI), and phosphodiesterase I were purchased from Sigma-Aldrich (St. Louis, MO, USA). *N*^6^-Methyl-2′-deoxyadenosine (6mA) and 5-methyl-2′-deoxycytidine (5mdC) were purchased from Berry & Associates (Dexter, MI, USA). DNase I, calf intestine alkaline phosphatase (CIAP), and TB Green Premix Ex *Taq*II (Tli RNaseH Plus) were purchased from Takara Biotechnology Co., Ltd. (Dalian, China). NEBNext Multiplex Oligos for Illumina (Index Primers Set 1) were purchased from New England Biolabs (Ipswich, MA, USA). Chromatography-grade methanol was purchased from Merck (Darmstadt, Germany). All other solvents and chemicals were of analytical grade.

### Cell Culture

The *E. coli* strains K12 MG1655 and SCS110 were cultured in 250 mL of LB medium at 37 °C for 12 h. *E. coli* cells were harvested by centrifugation at 10 000*g* for 5 min, washed three times with phosphate-buffered saline (PBS), and stored at −20 °C. HepG2 human liver carcinoma cells were cultured in Dulbecco’s modified Eagle’s medium (DMEM) at 37 °C under a 5% CO_2_ atmosphere. The medium was supplemented with 10% fetal bovine serum, 100 U/mL penicillin, and 100 μg/mL streptomycin (Gibco; Waltham, MA, USA).

### DNA Extraction

*E. coli* DNA was extracted using an Ezup column bacteria genomic DNA purification kit (Sangon Biotech, Shanghai, China) according to the manufacturer’s recommended procedures. Genomic DNA of HepG2 cells was extracted using a tissue DNA kit (Omega Bio-Tek Inc.; Norcross, GA, USA). Mitochondrial DNA of HepG2 cells was extracted as previously described with minor modifications.^37^ Briefly, HepG2 cells were harvested and washed once in a PBS buffer. Mitochondrial DNA was extracted using a high-purity plasmid DNA kit with RNA removal by RNase treatment (Tsingke Biotech; Beijing, China) according to the manufacturer’s recommended procedures. The extracted DNA was eluted in 200 μL of H_2_O and further enriched by 0.4× KAPA Pure Beads (Roche). The obtained DNA was stored at −20 °C until use.

### Enzymatic Digestion of DNA

A 30 μL mixture containing 10 ng of DNA, 4 U of DNase I, 0.002 U of phosphodiesterase I, 180 U of S1 nuclease, 15 U of CIAP, and 3 μL of reaction buffer (50 mM Tris-HCl, pH 7.0, 10 mM NaCl, 1 mM MgCl_2_, and 1 mM ZnSO_4_) was incubated at 37 °C for 3 h.^38,39^ The samples were extracted with chloroform twice. The aqueous layer was collected, lyophilized, and reconstituted in water for LC-ESI-MS/MS analysis.

### LC-ESI-MS/MS Analysis

LC-ESI-MS/MS analysis was performed on an AB 3200 QTRAP mass spectrometer (Applied Biosystems; Foster City, CA, USA) with an electrospray ionization (ESI) source. Data acquisition and processing were performed using AB SCIEX Analyst 1.5 software (Applied Biosystems; Foster City, CA, USA). The LC separation was performed on a Shimadzu VP-ODS column (250 mm × 2.1 mm i.d., 5 μm) with a flow rate of 0.2 mL/min at 35 °C. Formic acid in water (0.1%, solvent A) and methanol (solvent B) were employed as mobile phases. A gradient of 0–5 min 5% B, 5–20 min 5–70% B, 20–28 min 70% B, and 28–43 min 5% B was used. The mass spectrometer was operated in the positive ESI mode. The nucleosides were monitored in multiple-reaction monitoring (MRM) mode, and the detailed mass spectrometry parameters are listed in Table S2.

### Preparation of 6mA-Containing DNA

The 314-bp and 328-bp duplex DNAs were prepared by PCR amplification by using the pUC19 plasmid as the template. The PCR solution (50 μL) included 0.1 ng of pUC19 plasmid DNA, 25 μL of Q5 high-fidelity master mix (New England Biolabs), 1 μL each of the forward and reverse primers (10 μM), and 13 μL of H_2_O (sequences of primers are listed in Table S4). The PCR amplification program started at 98 °C for 30 s, followed by 30 cycles at 98 °C for 10 s, 60 °C for 30 s, and 72 °C for 30 s, and ended with 1 cycle at 72 °C for 5 min. The PCR products were separated by agarose gel and then purified using an agarose gel extraction kit (Zymo Research). As for the preparation of 6mA-containing DNA, 1 μg of 314-bp or 328-bp duplex DNA was incubated with 8 U of *dam* methyltransferase (New England Biolabs) in a 50 μL reaction buffer (50 mM Tris-HCl, pH 7.5, 5 mM β-mercaptoethanol, 10 mM EDTA, and 1.6 mM SAM) at 37 °C for 2 h (Figure S7). The resulting DNA was purified using an Oligo Clean & Concentrator Kit (Zymo Research). The sequences of 314-bp DNA and 328-bp DNA are provided in Table S1.

### Expression and Purification of ABE8e Protein

The full-length sequence of ABE8e was cloned into the pET-49b plasmid (pET-49b-ABE8e), which was transformed into the *E. coli* BL21(DE3) pLysS strain. The pET-49b-ABE8e encodes a glutathione *S*-transferase (GST) tag and the human rhinovirus 3C protease (HRV 3C) site. The *E. coli* cells transformed with pET-49b-ABE8e were grown in LB medium (tryptone 10 g/L, yeast extract 5 g/L, NaCl 10 g/L) supplemented with kanamycin (10 μg/mL) and chloramphenicol (10 μg/mL) at 37 °C under shaking at 180 rpm. Isopropyl-β-d-thiogalactoside (IPTG) was added to the medium to a final concentration of 0.5 mM when the OD_600_ of the *E. coli* cell suspension reached 0.6. The expression of the GST-ABE8e fusion protein was induced for 12 h at 18 °C under shaking at 180 rpm. The *E. coli* cells were then harvested by centrifugation at 10 000*g* for 5 min and lysed by sonication, followed by centrifugation at 12 000*g* and 4 °C for 10 min. The resulting supernatant was incubated with glutathione Sepharose 4B beads according to the manufacturer’s protocol. After digestion with HRV 3C protease (Sangon; Shanghai, China), the protein was further purified with a size-exclusion column (Millipore, Darmstadt, Germany) equilibrated with a storage buffer containing 50 mM tris-HCl (pH 7.5), 40% glycerol, and 0.5 mM dithiothreitol and stored at −80 °C before use. The purity of the protein was determined by SDS-PAGE (Figure S1), and the concentration of the protein was quantified using a BCA protein assay kit (Beyotime; Shanghai, China).

### Evaluation of the Deamination of Adenine/6mA in DNA by ABE8e

The synthesized 60-mer single A-DNA, single 6mA-DNA, or 314-bp duplex DNA (10 ng for each) was incubated with ABE8e (2.8 μM) in a 10 μL reaction buffer (50 mM Tris-HCl, pH 7.5, and 10 mM DTT) at 37 °C for different time intervals. Duplex DNA was first denatured by heating at 95 °C for 10 min in 10% DMSO (v/v) solution and chilling on ice for 5 min before the deamination reaction. The deamination reaction was quenched by heating at 95 °C for 10 min. The resulting DNA was purified with an Oligo Clean & Concentrator Kit (Zymo Research), and the deamination of adenine was examined with an endonuclease V (Endo V, New England Biolabs) cleavage assay or colony sequencing. As for the Endo V cleavage, the resulting DNA was incubated with 5 U of Endo V in 1× NEBuffer 4 at 37 °C for 1 h followed by polyacrylamide gel electrophoresis analysis. The gel was visualized using a Tanon fluorescence imager (Shanghai, China). As for colony sequencing, the resulting DNA was used as a template for PCR amplification. The PCR solution (25 μL) included 10 μL of deaminated DNA, 12.5 μL of TSINGKE Master Mix (Tsingke Biotech; Beijing, China), 1 μL each of the forward and reverse primers (10 μM), and 0.5 μL of H_2_O (sequences of primers are listed in Table S4). The amplification program started at 98 °C for 30 s, followed by 25 cycles at 98 °C for 10 s, 60 °C for 30 s, and 72 °C for 1 min, and ended with 1 cycle at 72 °C for 5 min. The PCR product was ligated and cloned using the pClone007 Blunt Simple Vector System (Tsingke Biotech) following the manufacturer’s instructions. Individual colonies were picked, lysed in TE buffer, amplified by PCR using M13 forward and reverse primers, and then sequenced using an ABI3700 (Applied Biosystems; Foster City, CA, USA). A total of 53 positive colonies for each sample were picked and subjected to Sanger sequencing.

### Steady-State Kinetics Study

A series of different amounts of 60-mer single A-DNA or single 6mA-DNA ranging from 0.1 to 5 μM were incubated with ABE8e (2.8 μM) in a 10 μL reaction buffer (50 mM Tris-HCl, pH 7.5, and 10 mM DTT) at 37 °C for 30 min. The reactions were quenched by heating at 95 °C for 10 min. The resulting DNA samples were digested and analyzed by LC-ESI-MS/MS.

The relative reaction velocity (*v*) was calculated from the ratio of the deaminated product (*I*_D_) over the undeaminated product (*I*_U_) plus the deaminated product (*I*_D_) as follows: *vt* = *I*_D_/(*I*_U_ + *I*_D_), where *t* represents the reaction time. The apparent *K*_M_ and *V*_max_ values were obtained from linear regression analysis of the Michaelis–Menten equation using the data points at different DNA concentrations in three independent experiments according to a previously described method.^40^ The enzymatic efficiency (*k*_cat_/*K*_m_) was used to describe the selectivity of ABE8e in deaminating A or 6mA.

### Library Construction for DM-seq

Genomic DNA was fragmented to obtain 300 to 500 bp fragments by using a JY92-II N ultrasonic homogenizer (Scientz) with the following settings: 130 W peak incident power for 48 cycles (1 cycle = 5 s on and 5 s off). The mixture (50 μL) of fragmented genomic DNA (10 ng) and spike-in DNA (328-bp 6mA-DNA, 5 pg) was end-repaired and dA-tailed using the Hieff NGS Fast-Pace End Repair/dA-Tailing Module (YEASEN Biotechnology Co., Ltd.; Shanghai, China) according to the manufacturer’s recommended protocol. As for the adapter ligation, 60 μL of end-repaired DNA, 5 μL of custom adapter (15 μM), 5 μL of Fast-Pace DNA Ligase, and 30 μL of Fast-Pace DNA Ligation Enhancer (YEASEN Biotechnology; Shanghai, China) were incubated at 20 °C for 12 h in a 100 μL solution. The DNA was purified by 0.8× KAPA Pure Beads (Roche) to remove the excess adaptor. The purified DNA was then denatured by heating at 95 °C for 10 min in 10% DMSO (v/v) solution and chilling on ice for 5 min. The ABE8e deamination reaction was conducted in a 10 μL reaction mixture containing 10 ng of DNA, 50 mM Tris-HCl (pH 7.5), 10 mM DTT, and 2.8 μM ABE8e. After incubation at 37 °C for 4 h, the reaction was stopped by heating to 95 °C for 10 min. The resulting DNA was purified by 0.8× KAPA Pure Beads (Roche) to remove the DTT and ABE8e. The purified DNA was subjected to PCR amplification. The PCR solution (25 μL) included 10 μL of purified DNA, 12.5 μL of TSINGKE Master Mix (Tsingke Biotech), 1 μL of pre-P7 primer (10 μM), 1 μL of pre-P5 primer (10 μM), and 0.5 μL of H_2_O. The amplification program started at 98 °C for 30 s, followed by 4 cycles at 98 °C for 10 s, 60 °C for 30 s, and 72 °C for 1 min. The PCR products were subsequently purified using 0.8× KAPA Pure Beads (Roche), followed by the second round of PCR amplification. The PCR solution (50 μL) included 20 μL of purified DNA, 0.5 μL of Phusion Plus DNA polymerase (Thermo Fisher Scientific), 10 μL of 5× Phusion Plus buffer, 10 μL of 5× Phusion GC Enhance, 1 μL of P7 primer (15 μM), 1 μL of P5 primer (15 μM), and 7.5 μL of H_2_O. The amplification program started at 98 °C for 30 s, followed by 13 cycles at 98 °C for 30 s, 72 °C for 45 s, and 1 cycle at 72 °C for 5 min. The PCR products were purified using 0.8× KAPA Pure Beads (Roche) and agarose gel with the use of an agarose gel extraction kit (Zymo Research). Library quality was assessed on an Agilent Bioanalyzer 2100 system. The library was sequenced on an Illumina Hiseq platform, and 150-bp paired-end reads were generated. The DM-seq data have been deposited into the NCBI Gene Expression Omnibus (GEO) under accession number GSE194212.

### Data Analysis

FastQC (v0.11.8) software (https://www.bioinformatics.babraham.ac.uk/projects/fastqc/) was used for quality control analysis of the raw sequencing data (fastq format). The raw reads were trimmed to remove low-quality bases and adaptor sequences with Trim Galore (version 0.6.7) and Cutadapt (version 3.5 with Python 3.9.7). Trimmed reads were mapped to the reference genome of *E. coli* (NCBI accession: ASM584v2), human (GRCh38.p13), and human mitochondrion (NC_012920.1) by HIAST-3N.^41,42^ Only unique reads mapped to mitochondrial DNA were employed for the downstream analysis. The methylation rate of dA sites was extracted by Hisat-3n-table. For each site, the number of “A” bases was counted as 6mA sites (denoted as *NA*) and the number of “G” bases was counted as dA sites (denoted *NG*). The sequencing depth (defined as *N* = *NA* + *NG*) and coverage of samples were calculated using samtools (v1.9) software.

The spike-in DNA was used to calculate the deamination rate (DR) of dA and protection rate (PR) of 6mA. The sites of 124, 125, 265, and 266 in spike-in DNA were 6mA. The deamination rate of dA and protection rate of 6mA were calculated using the following equations:
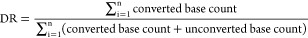

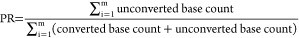
(m = 124, 125, 265, 266)

The methylation rates were calculated using the following equation,
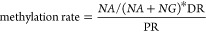


The 12 bp contexts upstream and downstream of 6mA sites were extracted and used for motif searching and identification with STREME (v5.4.1).

## Supporting Information Available

Experimental conditions for nitrite treatment and sequencing, a list of oligodeoxynucleotides used in the study, and an evaluation of DM-Seq results (PDF)
