# Supplementary material for: Single-Nucleotide Resolution Mapping of N6-Methyladenine in Genomic DNA
Source: ACS Cent Sci. 2023 Aug 28;9(9):1799–809. doi: 10.1021/acscentsci.3c00481 (PMC10540296; doi:10.1021/acscentsci.3c00481)
Supplement: Supplementary file 1 — oc3c00481_si_001.pdf [file oc3c00481_si_001.pdf]

Online Supporting Information for

"Single-nucleotide resolution mapping of *N*^{6}-methyladenine in genomic DNA"

Cheng-Jie Ma[1,2,3,†], Gaojie Li[4,5,†], Wen-Xuan Shao[1,2,†], Yi-Hao Min,[1,2] Ping Wang[4,5], Jiang-Hui Ding[1,2], Neng-Bin Xie[1,2], Min Wang[1,2], Feng Tang[3], Yu-Qi Feng[1,2], Weimin Ci[4,5,*], Yinsheng Wang[3,*], Bi-Feng Yuan[1,2,*]

[1] *School of Public Health, Department of Radiation and Medical Oncology, Zhongnan Hospital of Wuhan University, Wuhan University, Wuhan 430071, China.*

[2] *Sauvage Center for Molecular Sciences, Department of Chemistry, Wuhan University, Wuhan 430072, China.*

[3] *Department of Chemistry, University of California, Riverside, Riverside, California 92521-0403, United States.*

[4] *Key Laboratory of Genomics and Precision Medicine, and China National Center for Bioinformation, Beijing Institute of Genomics, Chinese Academy of Sciences, Beijing 100101, China.*

[5] *University of Chinese Academy of Sciences, Beijing 100049, China.*

[†]These authors contributed equally to this study

*Corresponding authors: Weimin Ci, Yinsheng Wang, Bi-Feng Yuan.

E-mail: ciwm@big.ac.cn, Yinsheng.Wang@ucr.edu, bfyuan@whu.edu.cn

Table of Contents:

**Contents**	**Pages**
Nitrite treatment and sequencing.	**S3**
**Table S1.** Sequences of oligonucleotides.	**S4**
**Table S2.** The mass spectrometry parameters for the analysis of nucleosides.	**S5**
**Table S3.** Evaluation of the deamination rate of dA in different sequence context of the 314-bp 6mA-DNA by colony sequencing.	**S6**
**Table S4.** Primers for PCR.	**S7-S8**
**Table S5.** Sequencing data of the spike-in DNA in different samples by DM-seq.	**S9**
**Table S6.** Sequencing data by DM-seq.	**S10**
**Table S7.** 6mA sites commonly identified in HepG2 mtDNA from three replicates by DM-seq method.	**S11**
**Figure S1.** SDS-PAGE analysis of the purified ABE8e protein.	**S12**
**Figure S2.** Quantitative evaluation of the Endo V cleavage assay using the mixture of 60-mer single I-DNA and 60-mer single A-DNA.	**S13**
**Figure S3.** Quantitative evaluation of the deamination of adenine in DNA by ABE8e using the mixture of 60-mer single A-DNA and 60-mer single 6mA-DNA.	**S14**
**Figure S4.** Evaluation of the deamination of adenine in the 60-mer single A-DNA by ABE8e at different times (0, 1, 5, 10, and 30 min).	**S15**
**Figure S5.** Evaluation of the normal nucleosides in DNA upon ABE8e treatment by LC-ESI-MS/MS.	**S16**
**Figure S6.** Evaluation of the resistance of 6mA to ABE8e.	**S17**
**Figure S7.** Preparation of 314-bp 6mA-DNA using *dam* methyltransferase.	**S18**
**Figure S8.** Colony sequencing using 314-bp 6mA-DNA after ABE8e treatment.	**S19**
**Figure S9.** Evaluation of the quantitative capability of DM-seq using synthesized DNA.	**S20**
**Figure S10.** Evaluation of the identification of 6mA sites in complex sequence context.	**S21**
**Figure S11.** Evaluation of the nitrite sequencing.	**S22**
**Figure S12.** Evaluation of the levels of dA, dI and 6mA in DNA from wild-type *E. coli* strain K12 (Dam+) or *dam* deficient *E. coli* strain SCS110 (Dam-) after ABE8e treatment by LC-ESI-MS/MS analysis.	**S23**
**Figure S13.** The schematic illustration of library preparation for DM-seq.	**S24**
**Figure S14.** The representative view of 6mA sites between the region of 98809 to 98829 in genome of *dam* deficient *E. coli* strain SCS110.	**S25**
**Figure S15.** Circos plot showing the identified G6mATC motif from three replicates.	**S26**
**Figure S16.** Evaluation of the real-time qPCR amplification efficiencies.	**S27**
**Figure S17.** The proportion of the number of reads mapped to mtDNA over the number of total clean reads.	**S28**
**Figure S18.** Confirmation of the selected 6mA sites in mtDNA by Sanger sequencing.	**S29**

Nitrite treatment and sequencing

Nitrite treatment was carried out according to previously published procedures study.[1] Briefly, 1.0 μg of synthetic 314-bp A-DNA (Table S1) in 8 μL H_2O was first denatured by heating at 95ºC for 10 min, followed by chilling on ice for 5 min. The denatured DNA was then incubated with 15 μL of fresh 2.0 M sodium nitrite and 7 μL 10% acetic acid (v/v) at 37ºC for 5 h. The resulting DNA was purified by Oligo Clean & Concentrator kit (Zymo Research) and subjected to Sanger sequencing and colony sequencing.

Reference:

1. Li, X.; Guo, S.; Cui, Y.; Zhang, Z.; Luo, X.; Angelova, M. T.; Landweber, L. F.; Wang, Y.; Wu, T. P., NT-seq: a chemical-based sequencing method for genomic methylome profiling. *Genome Biol* **2022,** *23* (1), 122.

Table S1. Sequences of oligonucleotides.

**oligonucleotides used in this study**	
Sequence Names	Sequences
60-mer single A-DNA	5′- FAM-GTTCGGTGGCTCCGTCCGTGTTCACCTTTCTTTGT<br>CTGTGGGCGTTTTGGTTGCTCTTCG -3′
60-mer single 6mA-DNA	5′- FAM-GTTCGGTGGCTCCGTCCGTGTTC6mACCTTTCTTT<br>GTCTGTGGGCGTTTTGGTTGCTCTTCG -3′
60-mer single I-DNA	5′- FAM-GTTCGGTGGCTCCGTCCGTGTTCICCTTTCTTTGT<br>CTGTGGGCGTTTTGGTTGCTCTTCG -3′
60-mer dual 6mA-DNA	5′-TTCGTTTGTTCAACGATTAGCGCCTTCAA6mAA6mAAACTCC<br>TACTCCTCATTGTACCCATTCT-3’
25-mer product DNA	5′- ATCAACCCTTCCACTGCTTG-3′
314-bp A-DNA	5′-<br>TTCGTTTGTTCAACGATTAAAGTCCTACGTGATCTGAGTTCAGACCGGA<br>GTAATCCAGGTCGGTTTCTATCTACTTCAAATTCCTCCCTGTACGAAAGG<br>ACAAGAGAAATAAGGCCTACTTCACAAAGCGCCTTCCCCCGTAAATGAT<br>ATCATCTCAACTTAGTATTATACCCACACCCACCCAAGAACAGGGTTTGT<br>TAAGATGGCAGAGCCCGGTAATCGCATAAAACTTAAAACTTTACAGTCA<br>GAGGTTCAATTCCTCTTCTTAACAACATACCCATGGCCAACCTCCTACT<br>CCTCATTGTACCCATTCT-3′
314-bp 6mA-DNA	5′-<br>TTCGTTTGTTCAACGATTAAAGTCCTACGTG6mATCTGAGTTCAGACCG<br>GAGTAATCCAGGTCGGTTTCTATCTACTTCAAATTCCTCCCTGTACGAAA<br>GGACAAGAGAAATAAGGCCTACTTCACAAAGCGCCTTCCCCCGTAAAT<br>GATATCATCTCAACTTAGTATTATACCCACACCCACCCAAGAACAGGGTT<br>TGTTAAGATGGCAGAGCCCGGTAATCGCATAAAACTTAAAACTTTACAGT<br>CAGAGGTTCAATTCCTCTTCTTAACAACATACCCATGGCCAACCTCCTA<br>CTCCTCATTGTACCCATTCT-3′
328-bp 6mA-DNA	5′-<br>TTTACACTTTATGCTTCCGGCTCGTATGTTGTGTGGAATTGTGAGCGGA<br>TAACAATTTCACACAGGAAACAGCTATGACCATGATTACGCCAAGCTTG<br>CATGCCTGCAGGTCGACTCTAGAGG6mATCCCCGGGTACCGAGCTCG<br>AATTCACTGGCCGTCGTTTTACAACGTCGTGACTGGGAAAACCCTGGC<br>GTTACCCAACTTAATCGCCTTGCAGCACATCCCCCTTTCGCCAGCTGG<br>CGTAATAGCGAAGAGGCCCGCACCG6mATCGCCCTTCCCAACAGTTGC<br>GCAGCCTGAATGGCGAATGGCGCCTGATGCGGTATTTTCTCCT-3′
69-mer custom adaptor	5′-<br>GGTGCAGCCGCTATTAAAGGTACTTCACATTCGTTTGTTCAACGATTACT<br>GATAGTGCTCTTCCGATCT -3′
67-mer custom adaptor	5′-<br>pGATCGGAAGAGCACTATCAGCCTCATTGTACCCATTCTGCCATTCGTA<br>ATCCTTACGGTAACGCTAA -3′

Note: p, 5′-phosphorylation.

Table S2. The mass spectrometry parameters for the analysis of nucleosides. DP, declustering potential; EP, entrance potential; CE, collision energy; CXP, collision cell exit potential.

Analytes	Precursor ion	Product ion	DP/V	EP/V	CE/V	CXP/V
dA	252.2	136.1	34.0	5.0	21.0	4.0
dC	228.2	112.1	22.0	4.0	15.0	4.0
dG	268.2	152.1	32.0	4.0	16.0	5.0
dT	243.2	127.0	30.0	4.5	16.0	3.0
6mA	266.1	150.1	35.0	8.0	23.0	2.0
dI	253.2	137.0	32.0	4.0	16.0	5.0

Table S3. Evaluation of the deamination rates of dA in different sequence contexts of the 314-bp 6mA-DNA by colony sequencing.

Motif	AAA	AAT	AAC	AAG	TAA	TAT	TAC	TAG	CAA	CAT	CAC	CAG	GAA	GAT	GAC	GAG
Theoretical numbers	530	318	424	371	477	212	477	53	477	318	159	318	159	159	106	265
Found A-to-G conversion numbers	529	318	423	366	477	212	477	53	475	318	159	316	159	159	105	264
Deamination rate (%)	99.81	100	99.76	98.65	100	100	100	100	99.58	100	100	99.37	100	100	99.06	99.62

Table S4. Primers for PCR.

**oligonucleotides used in this study**	
Sequence Names	Sequences
314-A-forward primer	5′- TTCGTTTGTTCAACGATTA -3′
314-A-reverse primer	5′- AGAATGGGTACAATGAGG -3′
314-I-forward primer	5′- TTCGTTTGTTCGGCGGTTG -3′
314-I-reverse primer	5′- AGAACGGGCACAACGAGG -3′
328-A-forward primer	5′- TTTACACTTTATGCTTCC -3′
328-A-reverse primer	5′- AGGAGAAAATACCGCATCA -3′
Pre-P5 primer	5′- ACACTCTTTCCCTACACGACGCTCTTCCGATCTCGGCGGTTGCTGGTGGTGCT -3′
Pre-P7 primer	5′- GACTGGAGTTCAGACGTGTGCTCTTCCGATCTACGGGCACAACGAGGCCGAC -3′
P7-index primer (K12-1)	5′- CAAGCAGAAGACGGCATACGAGATAACGTATCATGACTGGAGTTCAGACGTGTGCTCTTCCGATCT -3′
P7-index primer (K12-2)	5′- CAAGCAGAAGACGGCATACGAGATAACTATGCATGACTGGAGTTCAGACGTGTGCTCTTCCGATCT -3′
P7-index primer (K12-3)	5′- CAAGCAGAAGACGGCATACGAGATACCAGTTCATGACTGGAGTTCAGACGTGTGCTCTTCCGATCT -3′
P7-index primer (SCS110-1)	5′- CAAGCAGAAGACGGCATACGAGATATGTACCTTTGACTGGAGTTCAGACGTGTGCTCTTCCGATCT -3′
P7-index primer (SCS110-2)	5′- CAAGCAGAAGACGGCATACGAGATATTCTGTGTTGACTGGAGTTCAGACGTGTGCTCTTCCGATCT -3′
P7-index primer (SCS110-3)	5′- CAAGCAGAAGACGGCATACGAGATATTCCTGCTTGACTGGAGTTCAGACGTGTGCTCTTCCGATCT -3′
P7-index primer (mt-1)	5′- CAAGCAGAAGACGGCATACGAGATATAGCTTGTTGACTGGAGTTCAGACGTGTGCTCTTCCGATCT -3′
P7-index primer (mt-2)	5′- CAAGCAGAAGACGGCATACGAGATAGGCTACAGTGACTGGAGTTCAGACGTGTGCTCTTCCGATCT -3′
P7-index primer (mt-3)	5′- CAAGCAGAAGACGGCATACGAGATACTTGTACTTGACTGGAGTTCAGACGTGTGCTCTTCCGATCT -3′

P5-universal primer	5′- AATGATACGGCGACCACCGAGATCTACACTCTTTCCCTACACGACGCTCTTCCGATCT -3′
GAPDH-forward primer	5′- CATCACCAAGGACAAGCAGAC -3′
GAPGH-reverse primer	5′- CCGCTCAGCACAAAGAACCC -3′
mtDNA-forward primer	5′- CGGAGTAATCCAGGTCGGTT -3′
mtDNA-reverse primer	5′- ATCATTTACGGGGGAAGGCG -3′

Table S5. Sequencing data of the spiked-in DNA in different samples by DM-seq.

Library name	A-to-G conversion (%)	6mA protection (%)
E. coli K12-1	94.1	97.9
E. coli K12-2	89.7	98.3
E. coli K12-3	89.5	97.7
E. coli SCS110-1	95.5	98.6
E. coli SCS110-2	95.2	98.0
E. coli SCS110-3	96.6	96.7
Human HepG2 mtDNA-1	95.4	97.7
Human HepG2 mtDNA-2	94.3	98.0
Human HepG2 mtDNA-3	89.6	98.6

Table S6. Sequencing data by DM-seq.

Library name	Raw reads	Clean reads	Mapped reads	Average depth
E. coli K12-1	24074556	18564371	13357721	280
E. coli K12-2	20855269	15885814	11282724	182
E. coli K12-3	24977926	19860304	14470791	257
E. coli SCS110-1	26615867	18295974	10957029	115
E. coli SCS110-2	23443715	17059847	11836116	137
E. coli SCS110-3	21550949	15652066	12682090	149
Human HepG2 mtDNA-1	24004991	9938076	4532411	2215
Human HepG2 mtDNA-2	26351835	10978598	5344840	1784
Human HepG2 mtDNA-3	24347486	14328917	8088916	1970

Table S7. 6mA sites commonly identified in HepG2 mtDNA from three replicates by DM-seq method.

Chr	Position	Strand	Peaks reported in Mol Cell, 2020, 78, 382-395
ChrMT	504	-	Yes
ChrMT	3572	-	Yes
ChrMT	3898	-	
ChrMT	3899	-	
ChrMT	5443	-	Yes
ChrMT	5446	-	Yes
ChrMT	5464	-	Yes
ChrMT	6241	-	Yes
ChrMT	6293	-	
ChrMT	6297	-	
ChrMT	7818	-	
ChrMT	7823	-	
ChrMT	10953	-	Yes
ChrMT	12096	-	Yes
ChrMT	12105	-	Yes
ChrMT	13645	-	Yes
ChrMT	14123	-	Yes

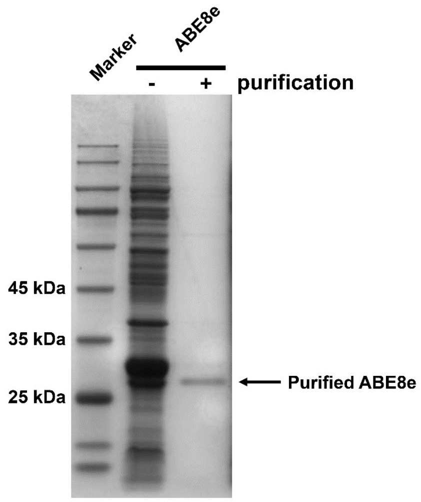

Figure S1. SDS-PAGE analysis of the purified ABE8e protein.

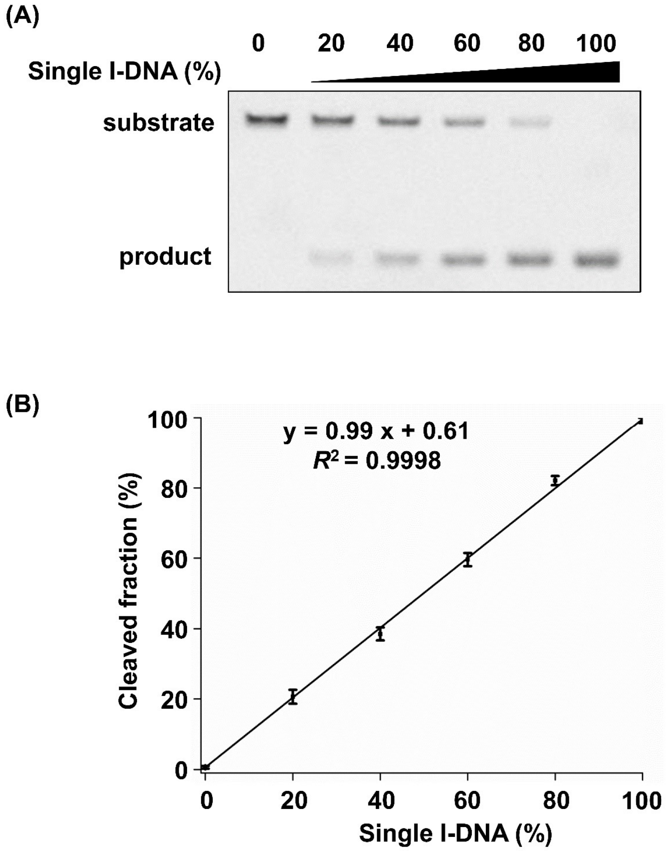

Figure S2. Quantitative evaluation of the Endo V cleavage assay using the mixture of 60-mer single I-DNA and 60-mer single A-DNA. (A) Single I-DNA and single A-DNA were mixed at different molar ratios (0, 20, 40, 60, 80, and 100% of single I-DNA) and then treated with Endo V at 37°C for 1 h. The resulting DNA was analyzed by polyacrylamide gel electrophoresis. (B) The cleaved fractions of single I-DNA were plotted against the percentage of single I-DNA. The slope of 0.99 indicated that the levels of single I-DNA in DNA mixture could be quantitatively obtained using the Endo V cleavage assay.

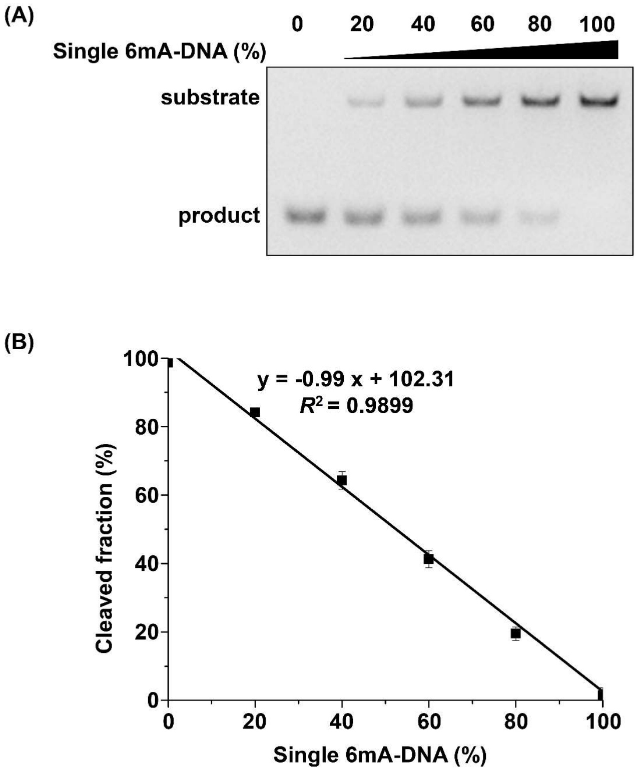

Figure S3. Quantitative evaluation of the deamination of adenine in DNA by ABE8e using the mixture of 60-mer single A-DNA and 60-mer single 6mA-DNA. (A) Analysis of the deamination of adenine by ABE8e with Endo V cleavage assay. Single A-DNA and single 6mA-DNA were mixed at different molar ratios (0, 20, 40, 60, 80, and 100% of single 6mA-DNA) and then treated with ABE8e at 37°C for 30 min. The resulting DNA was incubated with Endo V followed by polyacrylamide gel electrophoresis analysis. (B) The cleaved fractions of DNA were plotted against the percentage of single 6mA-DNA. The slope of -0.99 indicated that the levels of single 6mA-DNA in the DNA mixtures could be quantitatively obtained using the Endo V assay.

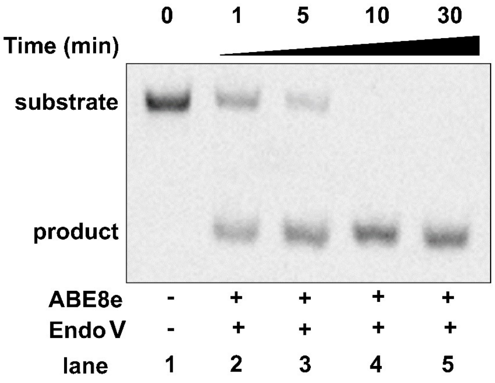

Figure S4. Evaluation of the deamination of adenine in the 60-mer single A-DNA by ABE8e at different times (0, 1, 5, 10, and 30 min). Endo V cleavage assay was used to evaluate the deamination of adenine.

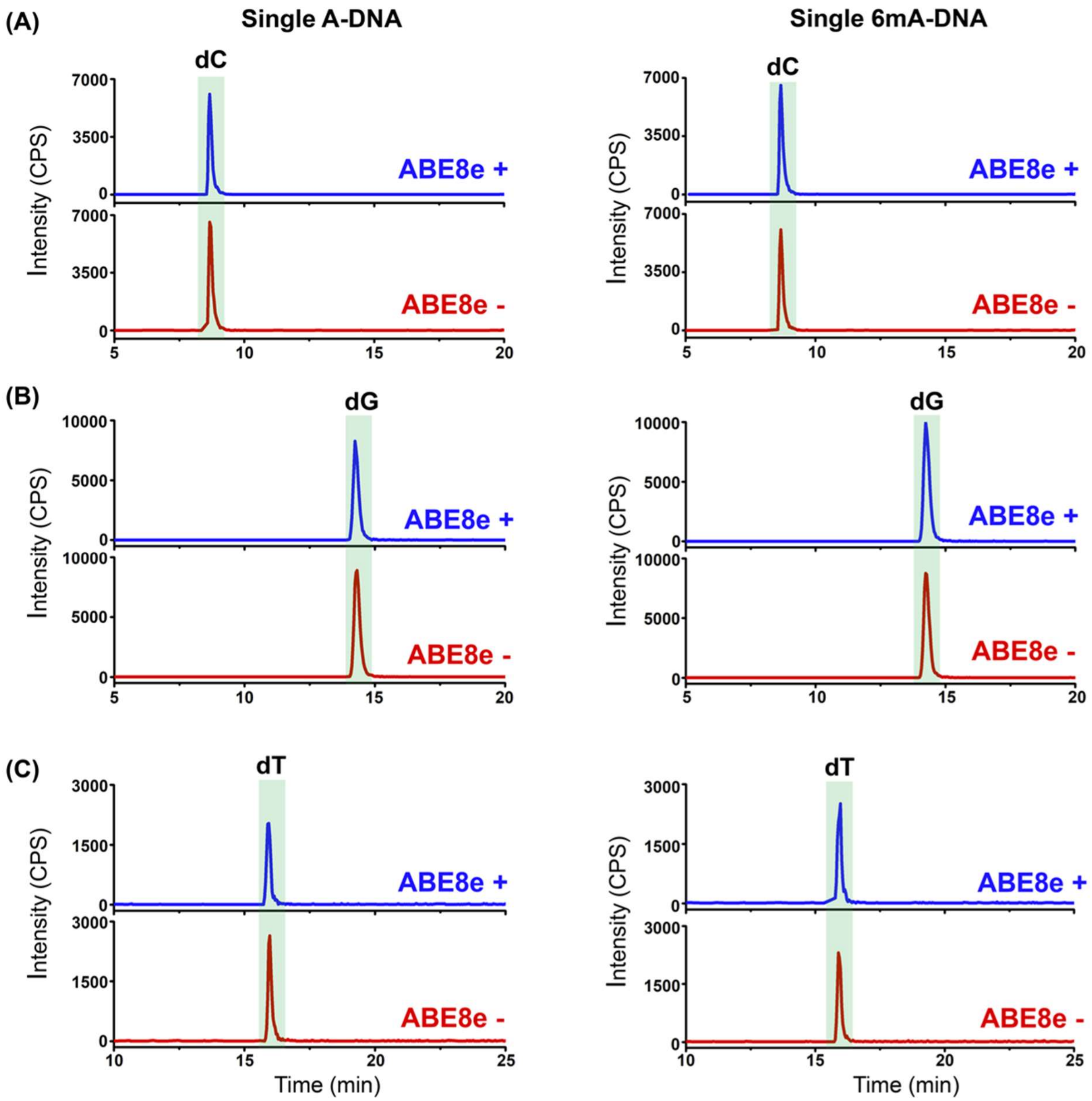

Figure S5. Evaluation of the normal nucleosides in DNA upon ABE8e treatment by LC-ESI-MS/MS. (A) Extracted-ion chromatograms of dC with or without ABE8e treatment in single A-DNA or single 6mA-DNA. (B) Extracted-ion chromatograms of dG with or without ABE8e treatment in single A-DNA or single 6mA-DNA. (C) Extracted-ion chromatograms of dT with or without ABE8e treatment in single A-DNA or single 6mA-DNA.

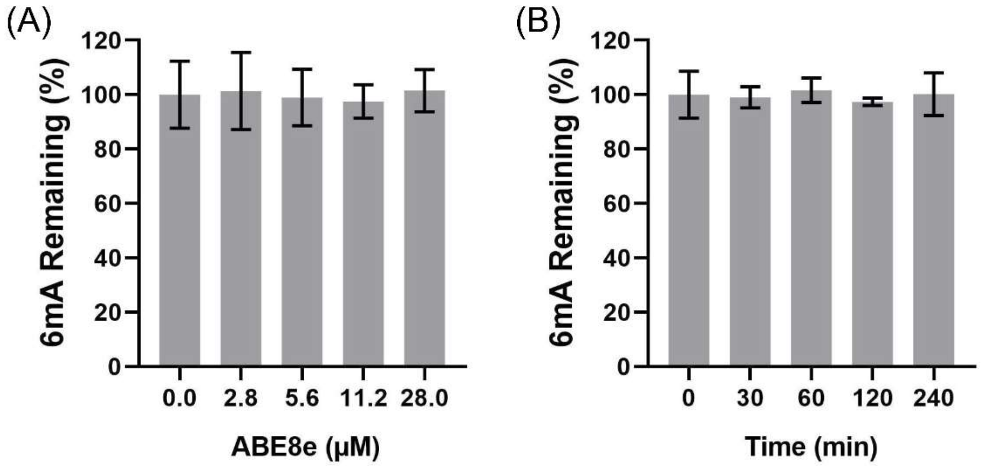

Figure S6. Evaluation of the resistance of 6mA to ABE8e. (A) LC-ESI-MS/MS analysis of the remaining percentage of 6mA upon incubation with different concentrations of ABE8e at 37ºC for 30 min. (B) LC-ESI-MS/MS analysis of the remaining percentage of 6mA upon incubation with of ABE8e (2.8 μM) at 37ºC for different time intervals. The 60-mer single 6mA-DNA was used for the evaluation.

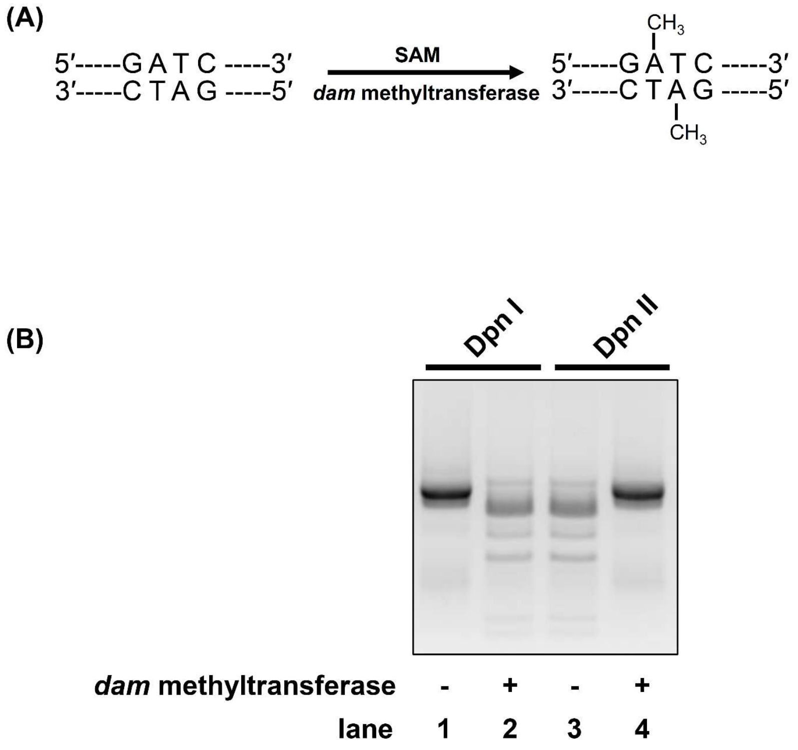

Figure S7. Preparation of 314-bp 6mA-DNA using *dam* methyltransferase. (A) A schematic diagram showing the methylation of 314-bp A-DNA by *dam* methyltransferase. *Dam* methyltransferase can methylate adenine at GATC site in the 314-bp A-DNA to generate the 314-bp 6mA-DNA. (B) Analysis of the prepared 314-bp 6mA-DNA by *Dpn* I and *Dpn* II digestion. *Dpn* I cleaves only when its recognition site is methylated, i.e., G6mATC site in DNA; but *Dpn* I cannot cleave the unmethylated GATC site in DNA. On the contrary, *Dpn* II cleaves only when its recognition site is unmethylated, i.e., GATC site in DNA; but *Dpn* II cannot cleave the methylated G6mATC site in DNA.

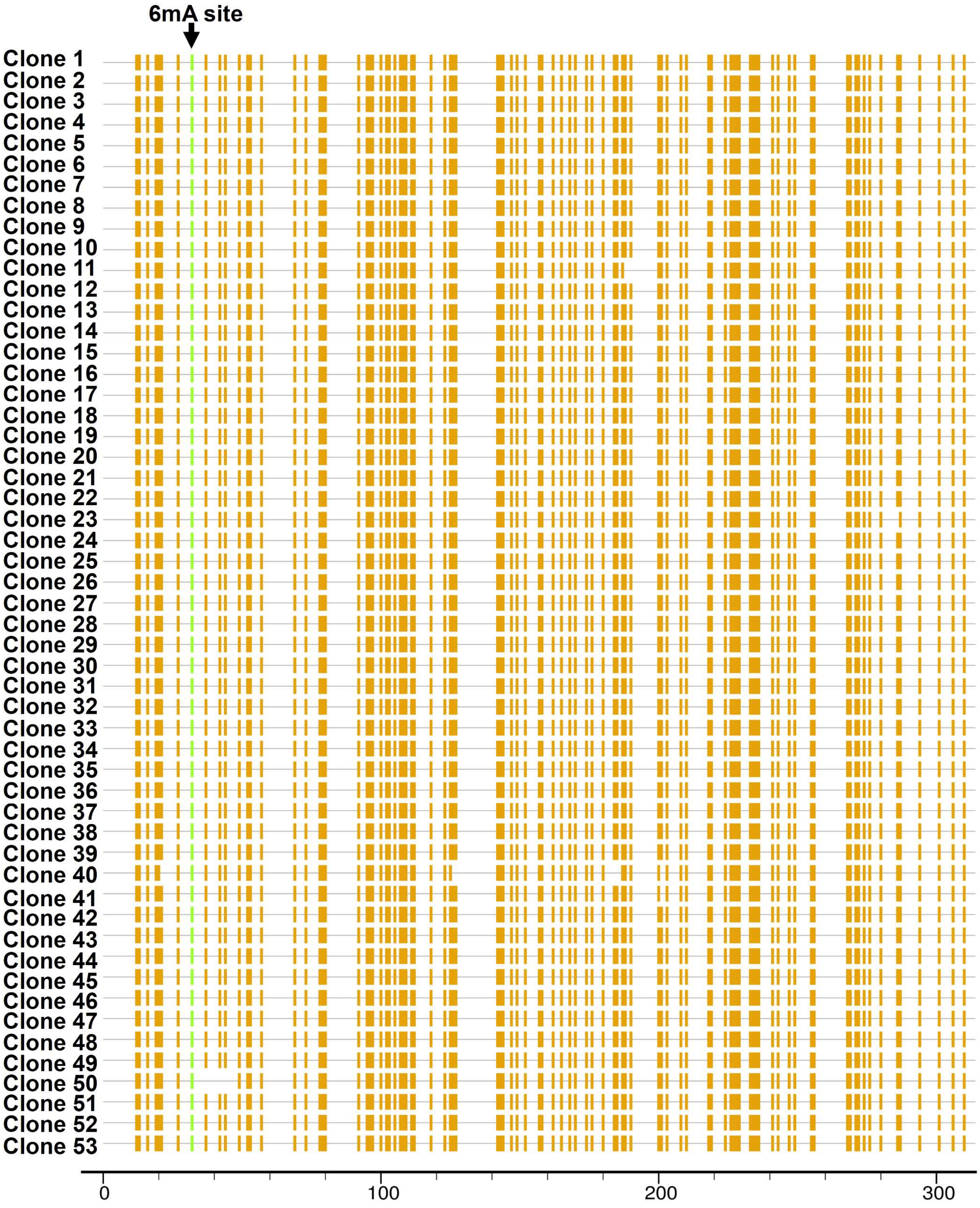

Figure S8. Colony sequencing using 314-bp 6mA-DNA after ABE8e treatment. Fifty-three colonies were picked for sequencing. The 6mA sites in all the 53 clones were read as A. A total of 4863 adenine sites out of 4876 (totally 92 adenines/strand × 53 clones = 4876 adenines) in 314-bp 6mA-DNA were read as G. The deamination rate was calculated to be 99.73% (4863/4876 × 100%). Green, read as A. Yellow, read as G.

(A)

6mA-DNA: GTTCGGTGGCTCCGTCCGTGTTC6mACCTTTCTTTGTCTGTGGGCGTTTTGGTTGCTCTTCG

A-DNA: GTTCGGTGGCTCCGTCCGTGTTC A CCTTTCTTTGTCTGTGGGCGTTTTGGTTGCTCTTCG

(B)

6mA-DNA (%)	Number of clones	Number of A reads	Number of G reads	Percentage of A reads
0%	134	0	134	0%
5%	98	3	95	3%
10%	105	10	95	9.5%
20%	105	23	82	22.2%
50%	102	54	48	54.5%
100%	138	136	2	98.4%

(C)

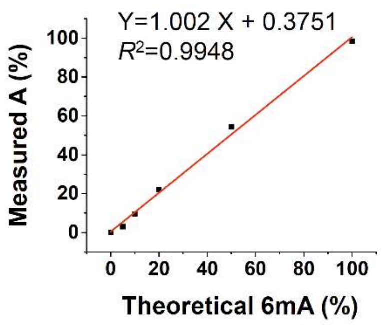

Figure S9. Evaluation of the quantitative capability of DM-seq using synthesized DNA. A 60-mer A-DNA was mixed with different percentages of 60-mer 6mA-DNA in the same sequence context (Table S1) and the readouts of A and G from the DM-Seq were evaluated. (A) The sequences of 60-mer A-DNA and 60-mer 6mA-DNA. (B) Colony sequencing showing the numbers of A reads and G reads from the mixtures with different 6mA percentages. (C) The measured percentages of A reads were plotted with the theoretical 6mA percentages.

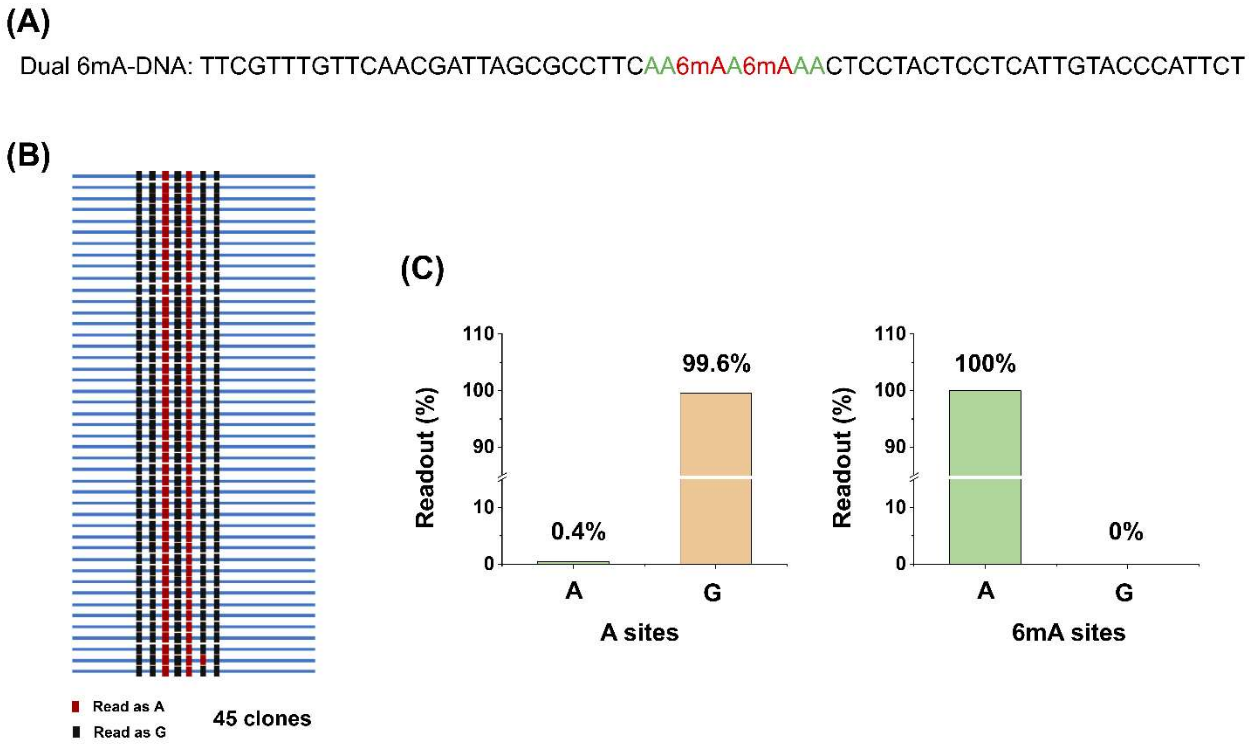

Figure S10. Evaluation of the identification of 6mA sites in complex sequence context. A 60-mer DNA containing two 6mA sites (dual 6mA-DNA) in the sequence context of AA6mAA6mAAA (Table S1) was synthesized and subjected to DM-seq analysis. (A) The sequence of 60-mer dual 6mA-DNA. (B) A schematic illustration showing the colony sequencing results from 45 clones. (C) The readouts from evaluated A sites and 6mA sites. All the A sites in this region were efficiently deaminated and read as G in sequencing (deamination rate of A being 99.6%). Both 6mA sites in this region resisted deamination and still read as A (deamination rate of 6mA being 0%).

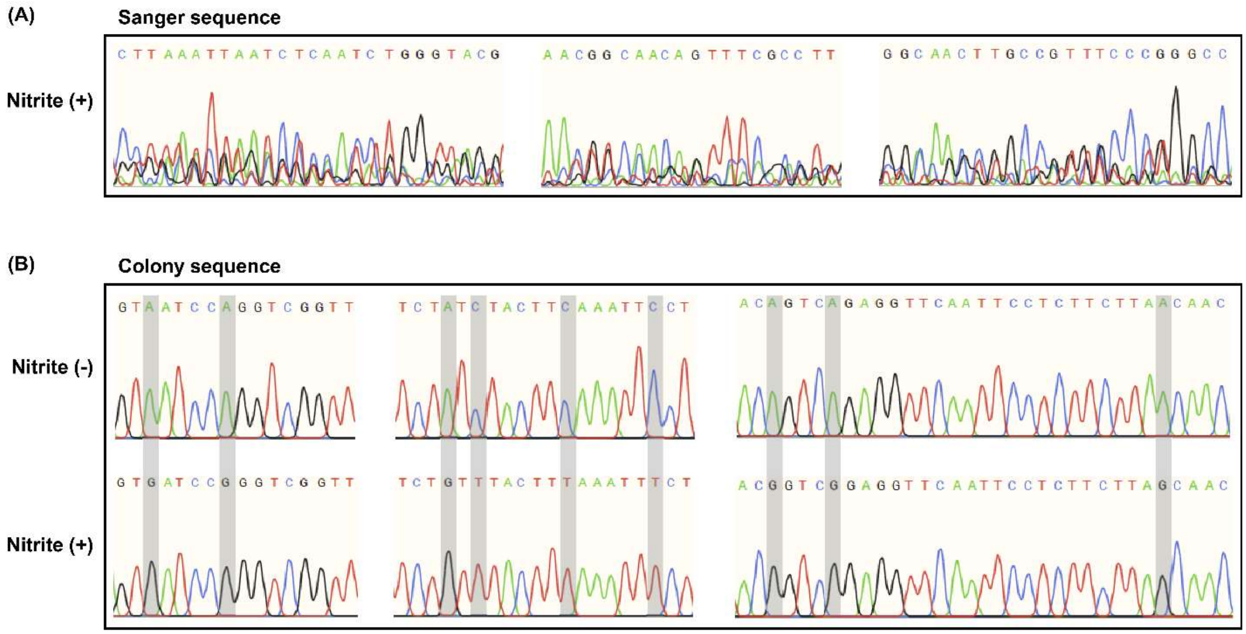

Figure S11. Evaluation of the nitrite sequencing. The 314-bp DNA (Table S1) was treated by nitrite according to previously described procedures followed by sequencing. (A) Direct Sanger sequencing results. (B) Representative colony sequencing results. Some A and C are deaminated and read as G and T, respectively.

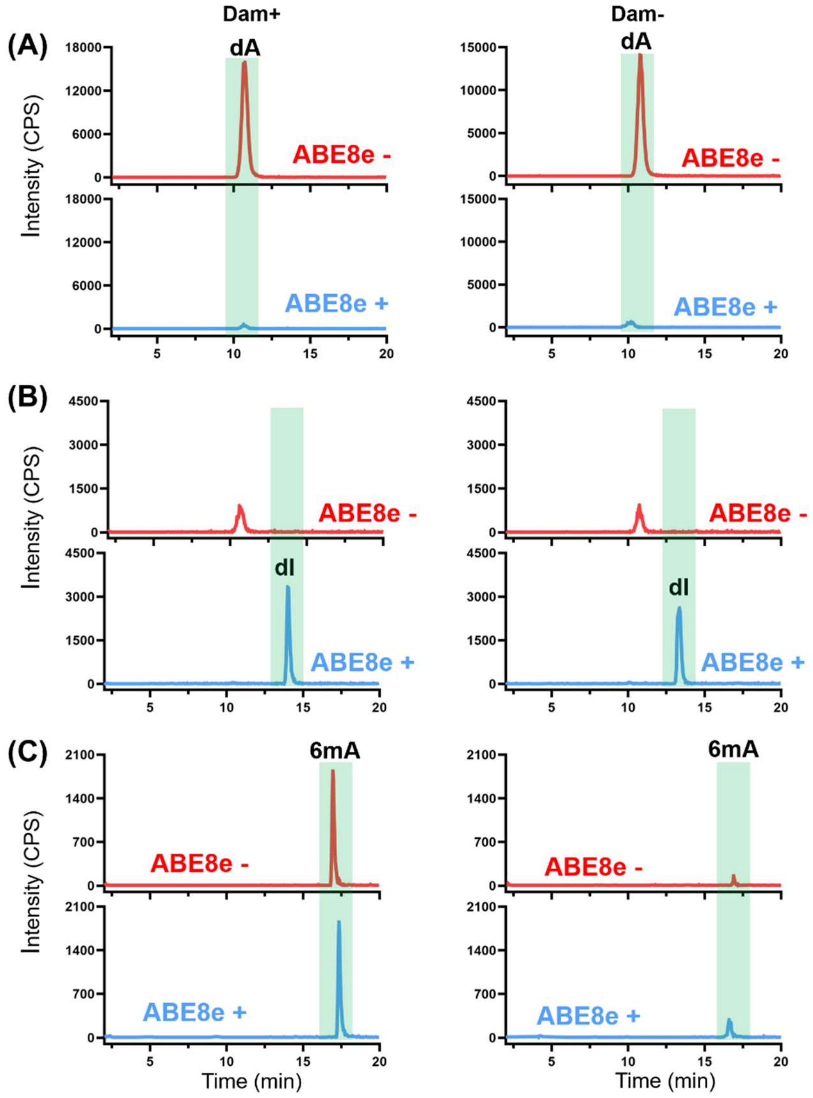

Figure S12. Evaluation of the levels of dA, dI and 6mA in DNA from wild-type *E. coli* strain K12 (Dam+) or *dam* deficient *E. coli* strain SCS110 (Dam-) upon ABE8e treatment by LC-ESI-MS/MS analysis. (A) Extracted-ion chromatograms of dA in DNA from Dam+ or Dam- *E. coli* cells with or without ABE8e treatment. (B) Extracted-ion chromatograms of dI in DNA from Dam+ or Dam- *E. coli* cells with or without ABE8e treatment. (C) Extracted-ion chromatograms of 6mA in DNA from Dam+ or Dam- *E. coli* cells with or without ABE8e treatment.

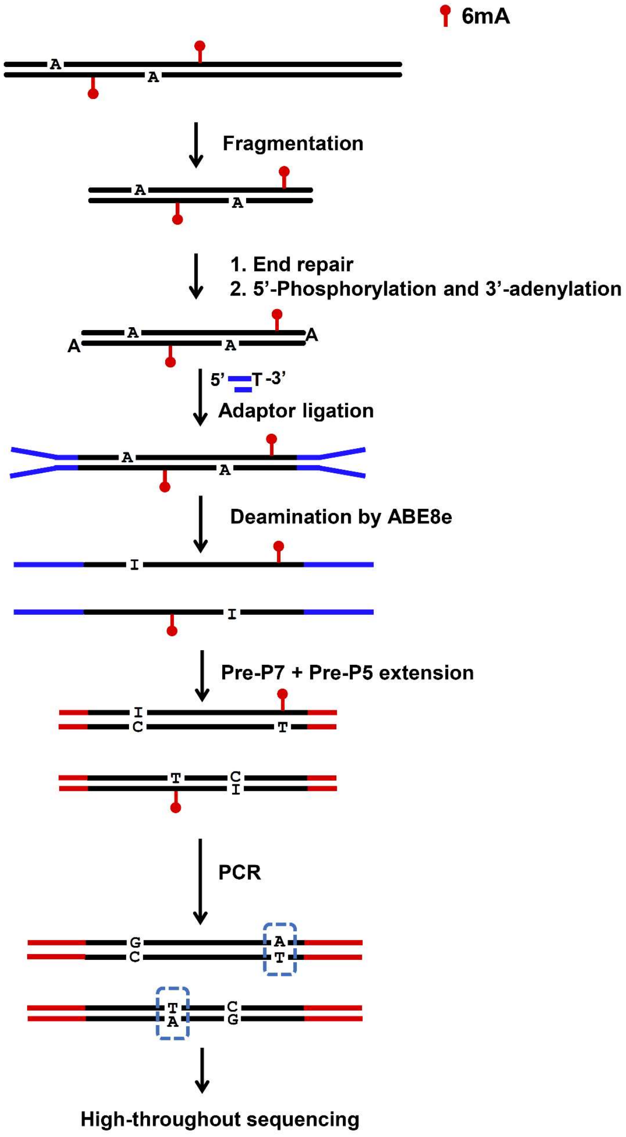

Figure S13. The schematic illustration of library preparation for DM-seq.

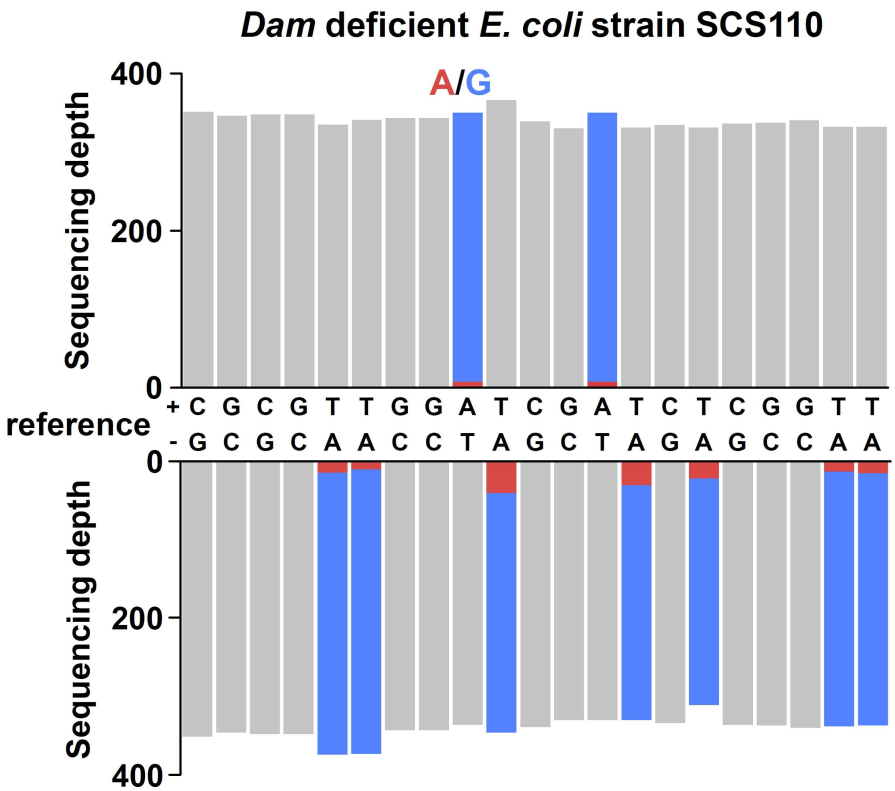

Figure S14. The representative view of 6mA sites between the region of 98809 to 98829 in genome of *dam* deficient *E. coli* strain SCS110. Red and blue columns represent the number of reads with A and G at given position, respectively.

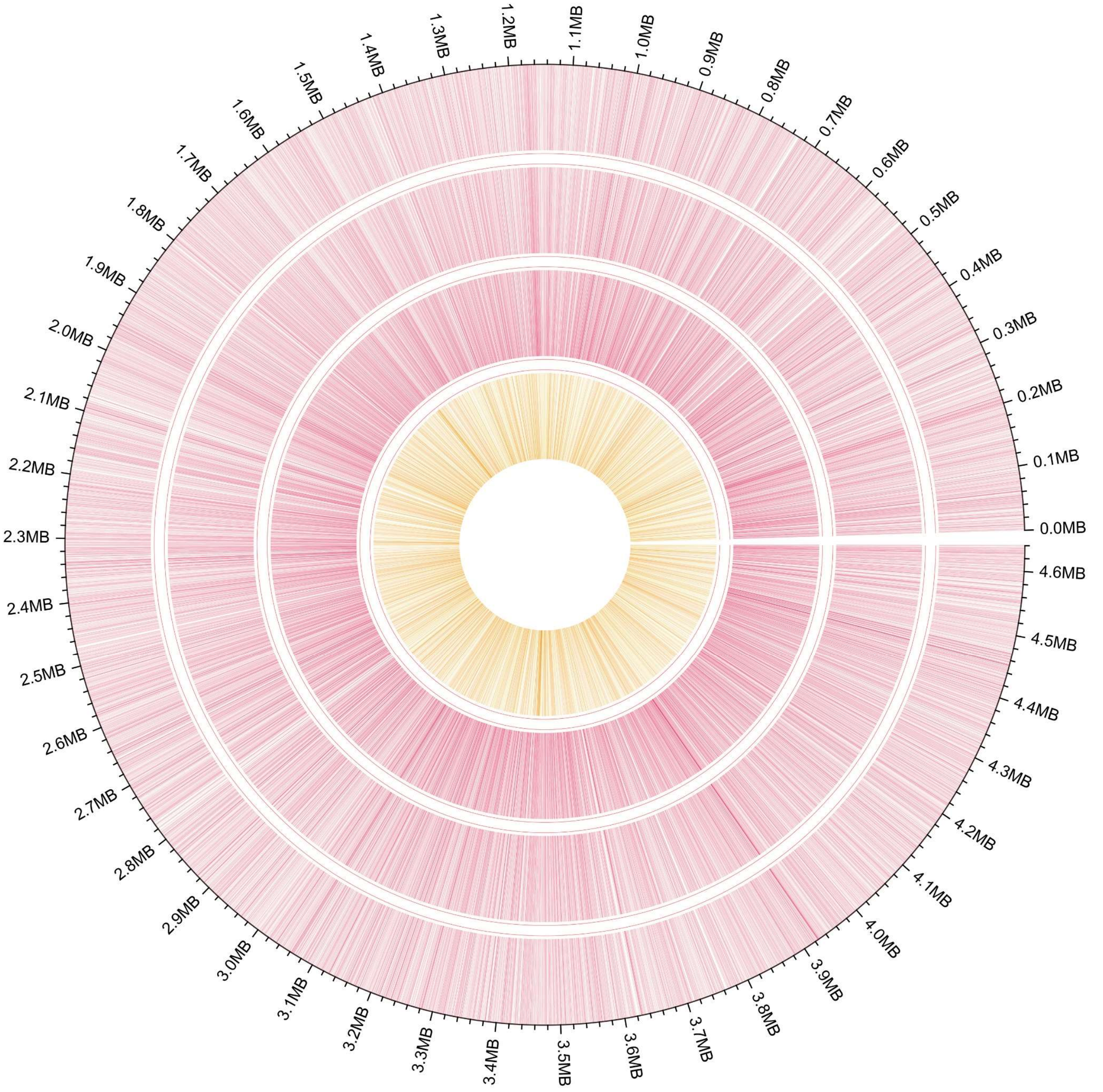

Figure S15. Circos plot showing the identified G6mATC motif from three replicates. Outer three circles with red bars represent the identified G6mATC motif from three replicates. Inner circle with yellow bars showing the theoretical sites of GATC in wild-type *E. coli* strain K12. The majority of the yellow lines (GATC sites) were red in all the outer three replicates, indicating consistent identification of 6mA.

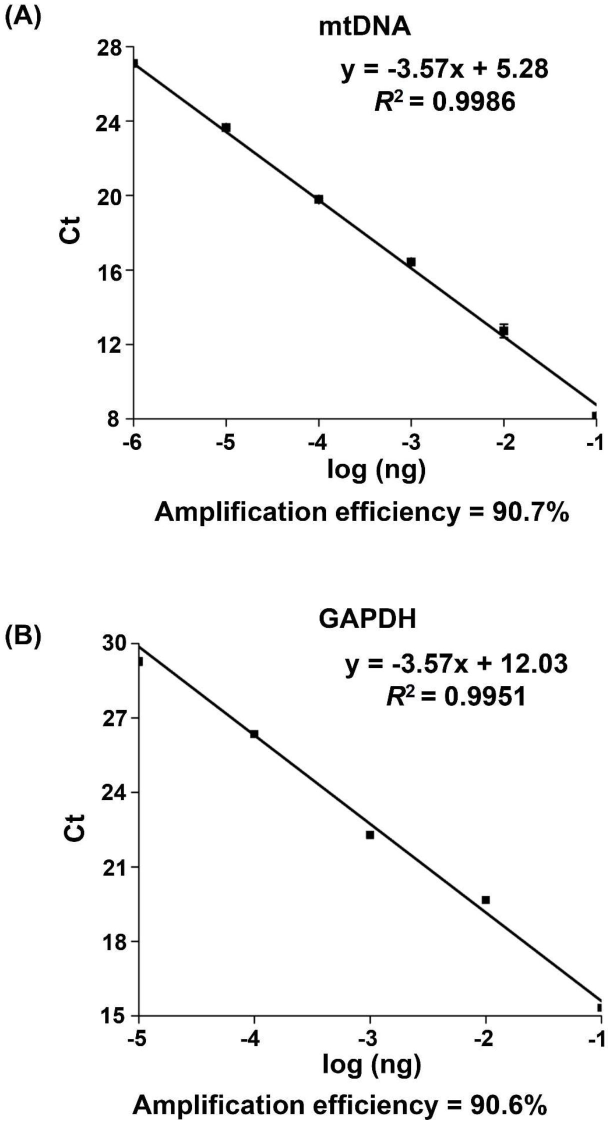

Figure S16. Evaluation of the real-time qPCR amplification efficiencies. (A) Standard curve for amplification of gene in mtDNA. (B) Standard curve for amplification of *GAPDH* in genomic DNA. The standard curves were constructed with using a series of amounts of DNA templates (0.001, 0.01, 0.1, 1, 10, and 100 ng) for real-time qPCR. The obtained amplification efficiencies of mtDNA and *GAPDH* were 90.7% and 90.6%, respectively.

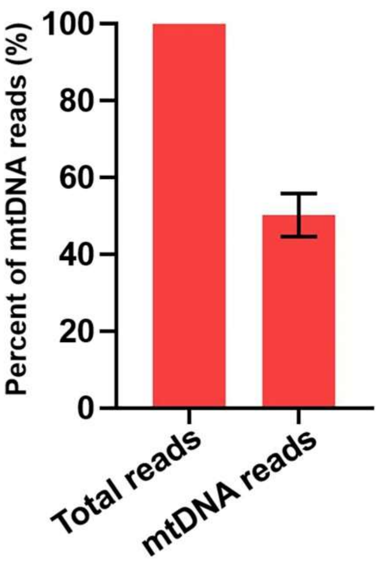

Figure S17. The proportion of the number of reads mapped to mtDNA over the number of total clean reads.

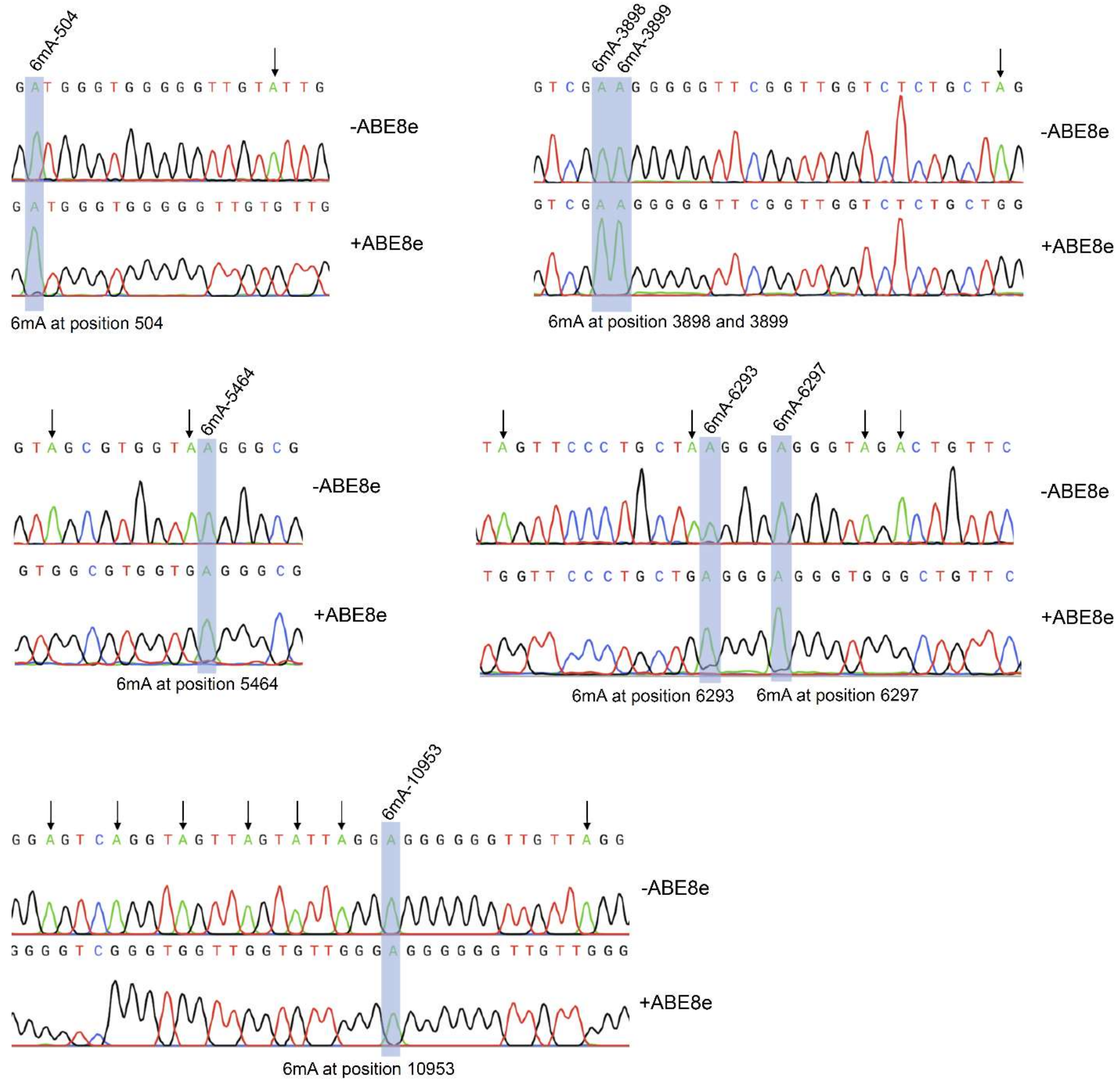

Figure S18. Confirmation of selected 6mA sites in mtDNA by Sanger sequencing. In Sanger sequencing, the 6mA sites identified in mtDNA by DM-seq were read as A, while other A sites were read as G.
